# Novel drug delivery strategies for antidepressant active ingredients from natural medicinal plants: the state of the art

**DOI:** 10.1186/s12951-023-02159-9

**Published:** 2023-10-27

**Authors:** Shun Yuan, Ting Ma, Ya-Nan Zhang, Ning Wang, Zulqarnain Baloch, Ke Ma

**Affiliations:** 1 Shandong University of Traditional Chinese Medicine, Jinan, 250355 People’s Republic of China; 2 Shandong Co-Innovation Center of Classic TCM Formula, Shandong University of Traditional Chinese Medicine, No 4655, University Road, Changqing District, Jinan, 250355 Shandong China; 3 Faculty of Life Science and Technology, Kunming University of Science and Technology, Kunming, 650500 Yunnan People’s Republic of China

**Keywords:** Novel Drug Delivery System, Natural medicinal plant, Depression, Novel drug delivery carriers, Novel drug delivery pathways

## Abstract

Depression is a severe mental disorder among public health issues. Researchers in the field of mental health and clinical psychiatrists have long been faced with difficulties in slow treatment cycles, high recurrence rates, and lagging efficacy. These obstacles have forced us to seek more advanced and effective treatments. Research has shown that novel drug delivery strategies for natural medicinal plants can effectively improve the utilization efficiency of the active molecules in these plants and therefore improve their efficacy. Currently, with the development of treatment technologies and the constant updating of novel drug delivery strategies, the addition of natural medicinal antidepressant therapy has given new significance to the study of depression treatment against the background of novel drug delivery systems. Based on this, this review comprehensively evaluates and analyses the research progress in novel drug delivery systems, including nanodrug delivery technology, in intervention research strategies for neurological diseases from the perspective of natural medicines for depression treatment. This provided a new theoretical foundation for the development and application of novel drug delivery strategies and drug delivery technologies in basic and clinical drug research fields.

**Graphical Abstract:**

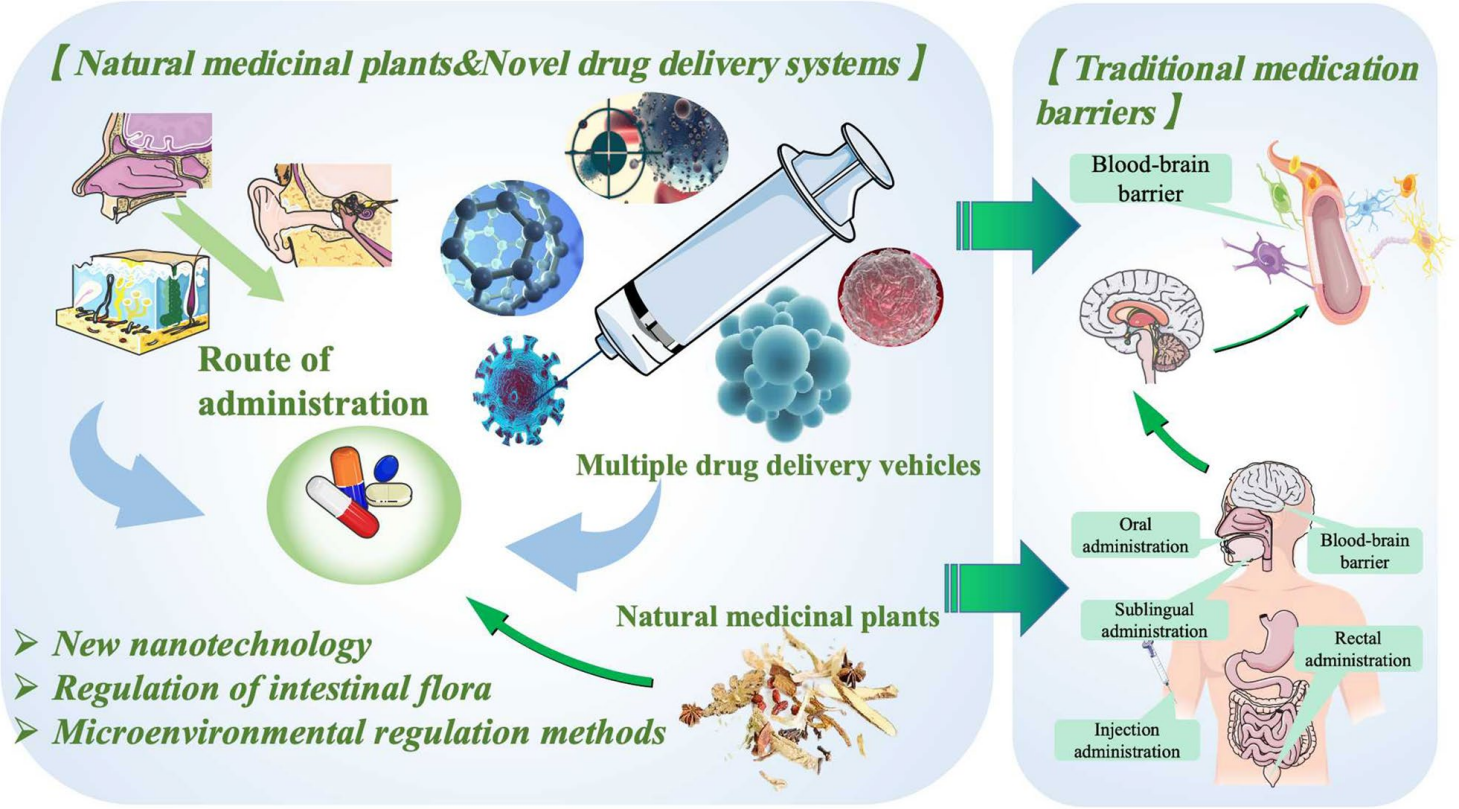

## Introduction

Depression is a widespread and potentially life-threatening neurological disorder that has garnered attention from researchers around the world. It not only has psychological manifestations involving emotions and cognition but also manifests neurological damage, such as decreased volition and cognitive impairment, and even physical symptoms, such as autonomic nervous system dysfunction. Moreover, depression often co-occurs with other psychiatric disorders, such as posttraumatic stress disorder, anxiety disorders, obsessive–compulsive disorder, and substance use disorders, and comorbidities, such as cardiovascular disease, diabetes, stroke, and cancer, frequently occur [1]. Depression has become a highly concerning public health issue on an international scale. However, due to the complex pathological mechanisms of neurological diseases, frontline health care professionals often face clinical difficulties such as complex adverse reactions and low treatment efficacy. As a result, scientists have started contemplating multiple possible treatment strategies. The combination of natural medicines and novel drug delivery systems (NDDSs) has entered our vision.

The attributes of natural medicines, possessing mechanisms of action that can intervene in multiple disease targets and pathways, have stimulated considerable interest among researchers in natural drug antidepressant therapies. Natural medications have the characteristics of fewer adverse reactions, significant therapeutic effects, and the ability to function in synergy with multiple components. Compared to traditional synthesized small-molecule drugs, natural drugs also have the advantages of abundant resources, low cost, and easy availability, making them an important source of new drug development. More than half of the new chemical entity drugs developed internationally are directly or indirectly derived from natural medications. Natural medications have enormous medical value and market potential.

The vigorous development of science and technology has brought breakthroughs in medical technology. The updating of the research content and paradigm in the field of drug delivery systems has solved multiple problems in drug bioavailability and stability, providing more possibilities for the treatment of clinical nervous system disorders. The breakthroughs in drug delivery routes and delivery vehicle research in the drug delivery system field have improved the efficacy of drugs used to treat nervous system diseases. The emergence of NDDSs has shown new clinical prospects in the formulation research of drugs. The development and maturity of drug delivery technology have provided impetus for the clinical transformation of many new drugs.

With the development of pharmaceuticals and the high pursuit of efficacy, the medical community has conducted further development and research on the enhancement of the absorption efficiency of active ingredients in natural medicines through the application of NDDS technology, which has resulted in notable therapeutic outcomes. Mature scientific research methods, such as nanotechnology, have significantly propelled the development of natural medicinal plant antidepressant treatment practices. Therefore, this review discusses interventions in neurological disease research from the perspective of novel drug delivery systems and involves exploring natural medicinal plant antidepressant strategies. The information in this review provides a novel theoretical foundation for studying new delivery strategies of natural medicinal plant active ingredients in the field of psychiatric research.

## Obstacles to traditional drug delivery methods and obstacles to BBB

Based on the evidence from modern medical systems, natural medicines encompass plant-, animal-, and mineral-derived drugs that possess pharmacological effects. Among these, plant-based medicines predominate in natural medicine, whereas animal, mineral, and other forms of natural medicines are used to a relatively lesser extent. Over time, various applied systematic theories have evolved in the field of natural medicine, including Chinese medicine, Indian Buddhist medicine, Islamic medicine, European traditional herbal medicine, South American ethnomedicine, and African ethnomedicine.

**Fig. 1:**
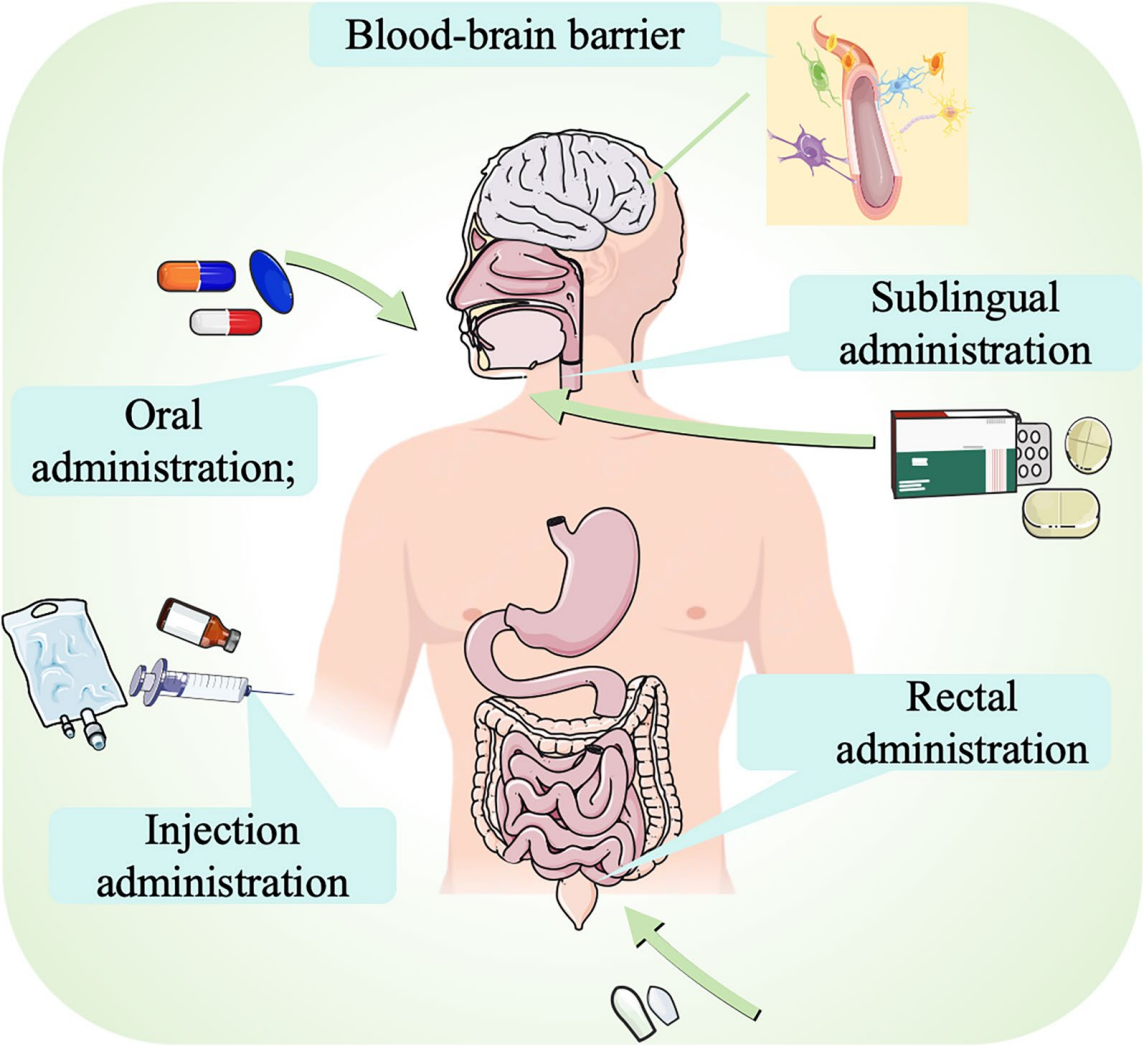
Traditional Drug Delivery and the Blood–Brain Barrier The obstacles to drug delivery methods for neurological diseases include the blood‒brain barrier and the limitations of traditional drug delivery systems such as oral administration, injection, sublingual administration, and rectal administration

The study of nervous system diseases in traditional Chinese medicine (TCM) has a long history; as early as the Shang and Zhou Dynasties in ancient China, there were records about brain disorders in oracle bone inscriptions, such as ‘King Wu was diagnosed due to headaches’ [2]. ‘*Huang Di Nei Jin*’ also contains numerous discussions on brain disorders [3, 4]. However, in the past, due to the use of traditional administration methods, Chinese medicine could not exert its desired therapeutic effect. At the same time, the existence of the blood–brain barrier (BBB) in the brain was an obstacle to the treatment of nervous system diseases [5] (Fig. 1).

The BBB, comprising several distinct cell types, such as brain capillary endothelial cells (BCECs), astrocytes, pericytes, and nerve cells, is a complex obstacle for drug delivery in the treatment of nervous system diseases. This barrier was initially identified by Paul Ehrlich in 1885 [6]. BCECs are the main component of the BBB responsible for selective permeability to small lipophilic molecules [7]. These cells tightly bind together to prevent paracellular drug transport across the BBB and contribute to the high transendothelial resistance (TEER) that limits the passive diffusion of external compounds between the brain and blood [8, 9]. Both pericytes and astrocytes play a supporting role in maintaining the structure and function of the BBB [10].

The BBB plays a crucial role in maintaining the internal environment of the nervous system by acting as a selectively permeable barrier that permits only small, nonpolar compounds weighing less than 400 Da to enter the brain. These compounds include ultrasmall molecules such as H_2_O, O_2_, and hydrophilic molecules [11]. Additionally, the BBB is adorned with specialized transport proteins, receptors, and other mechanisms, such as efflux transport proteins and ion-mediated channels, to facilitate the entry of vital components and metabolites into the brain [12].

The exact regulation of the homeostatic function of the central nervous system by the BBB not only ensures optimal neuronal performance but also shields neural tissue from harmful toxins and pathogens [13]. The existence of the BBB provides the CNS vasculature with the capacity to precisely govern the transfer of molecules, ions, and cells between the bloodstream and the CNS [14]. The highly limited barrier capacity of the BBB enables it to stringently regulate CNS homeostasis while simultaneously creating a hindrance to drug delivery to the CNS [15]. The BBB excludes 98% of small-molecule drugs and almost all large-molecule drugs, such as recombinant proteins, peptides, and antibodies [16]. When administered intravenously, it has been estimated that only 0.1% of therapeutic antibodies are able to enter the brain. This necessitates an elevation in drug concentrations in the bloodstream or prolonged administration to achieve therapeutic concentrations and target the disease effectively. However, resorting to these measures simultaneously raises the risk of systemic toxicity [17]. Apart from the aforementioned challenges faced in drug delivery, neurological disorders are characterized by lengthy prodromal periods, a scarcity of biomarkers, significant variability, and the occurrence of other disorders during their development [18]. Hence, addressing the arduous task of treating neurological disorders can be achieved only through symptomatic treatment, alongside intensified efforts in advancing neurological drugs and implementing innovative strategies to bypass the blood–brain barrier.

Traditional administration methods include oral administration, sublingual administration, rectal administration and injectable administration (Table 1). Oral administration is the most commonly utilized and safest method of drug delivery, offering unparalleled convenience and cost-effectiveness [19]. However, oral administration is hindered by the physicochemical properties of the drug itself, as well as the hepatic first-pass effect, the irritating effects of the drug on the gastric mucosa, and the impact of food present within the gastrointestinal tract [20]. Furthermore, the active ingredients of the drug may be compromised by digestive enzymes and gastric acids in the stomach and intestinal enzymes, resulting in a lower efficacy [21]. On the other hand, sublingual administration is limited by the small surface area of the oral mucosa. While injectable administration can deliver drugs rapidly to plasma and tissues, it also increases the risk of adverse effects [22].

**Table 1:** Traditional drug delivery methods

Drug delivery methods	Advantages	Disadvantages	References
Oral administration	Contains both solid and liquid dosage forms Noninvasive administration Simple and convenient	The absorption of the drug is strongly influenced by the first-pass effect in the liver and the destruction of digestive enzymes in the gastrointestinal tract Drugs cannot be orally administered to unconscious persons	[23, 24]
Sublingual administration	Includes sublingual and buccal routes, avoids the first-pass effect, easy to administer	Irritating to the oral mucosa Taste not preferred by patients	[25, 26]
Injection administration	Includes intravenous, intramuscular and subcutaneous routes Avoids the first-pass effect Precise control of blood concentration Can be given to unconscious patients	There is a risk of phlebitis, drug extravasation, and allergies The effect is related to changes in blood flow Taking stimulating drugs can cause pain	[27– 29]
Rectal administration	Can be used for patients who cannot swallow, vomit, or are unconscious Avoids the first-pass effect	Not convenient to use Drug absorption is slow or erratic Not accepted by the patient	[29–31, 65, 72, 75]

When applying TCM treatment techniques for neurological diseases, obstacles arise due to traditional drug delivery methods as well as barriers at the BBB, necessitating continuous efforts to improve drug delivery systems, enhance drug targeting capabilities, minimize off-target effects, and improve patient compliance [32]. As therapeutic drugs have progressed from small molecules to nucleic acids, peptides, proteins and even antibodies, our understanding of TCM has similarly advanced in stages. We have moved from viewing TCM as a monomeric entity to recognizing the active protein components of TCMs and from considering only the symptoms that drugs address to the specific targets that drugs regulate [33]. As drug application continues to advance and expand in scope, drug delivery technologies must also evolve in response to these new challenges. Drug delivery strategies and technologies are being adapted quickly to ensure they are able to meet the changing needs of drug delivery [34].

NDDSs represent a significant advancement in the field of drug delivery systems (DDSs) compared to conventional delivery methods [35]. These technologies are designed to regulate drug distribution in the body in terms of space, time, and dosage, ensuring optimal drug delivery. The overarching aim of NDDSs is to enhance drug utilization, improve drug efficacy, minimize toxicity, and reduce costs by delivering the right amount of medication to the right location at the appropriate time [36].

## The link between NDDSs and antidepressant treatment in Chinese medicine

Depressive neurosis belongs to the category of “depression syndrome” in traditional Chinese medicine. The classic Chinese medical text ‘*Danxi's Experiential Therapy: Six Depressions*’ proposed that “qi and blood in coordination, all diseases do not arise; when there is depression, various diseases arise”. In ancient times, medical experts often attributed depression to emotional discomfort and stagnant qi. They also emphasized the roles of disharmony between qi and blood, asthenic yin causing excessive pyrexia, and inadequate nourishment of the heart and mind in the onset of depression [37]. Modern medical practitioners are becoming more knowledgeable in their understanding of depression and acknowledging its complex pathology, which involves the brain, liver, gallbladder, heart, spleen, and kidneys. It is widely accepted that the etiology of depression cannot be generalized and may vary depending on factors such as age, sex, environment, and constitution [38].

It has been found that the goldenseal contained in forsythia has a unique antidepressant effect. Li Ruyue et al. conducted a study to investigate the potential pharmacodynamic effects and pharmacokinetic interactions of Guanye forsythia combined with sertraline in a rat corticosterone-induced depression model. Their findings showed that the combination of forsythia and sertraline significantly improved behavioral indicators and neurotransmitter levels in this rat model of depression [38]. Modern pharmacological studies have shown that saikosaponins, the active ingredients of Radix Bupleuri, potentiate the antidepressant-like effects of fluoxetine [39]. Flavonoids, which are the active ingredients in Radix Scutellariae Purging fire to remove trouble, are known to exhibit significant neuromodulator activities, such as having antidepressant and anxiolytic effects [40]. Research indicates that liquiritin can effectively reverse the behavioral changes observed in chronic stress and depression model rats, these findings suggest that liquiritin has antidepressant-like effects [41]. Curcumin exerts antidepressant-like effects by potentiating monoamine transmitter action through the inhibition of monoamine oxidase (MAO) [42].

**Fig. 2:**
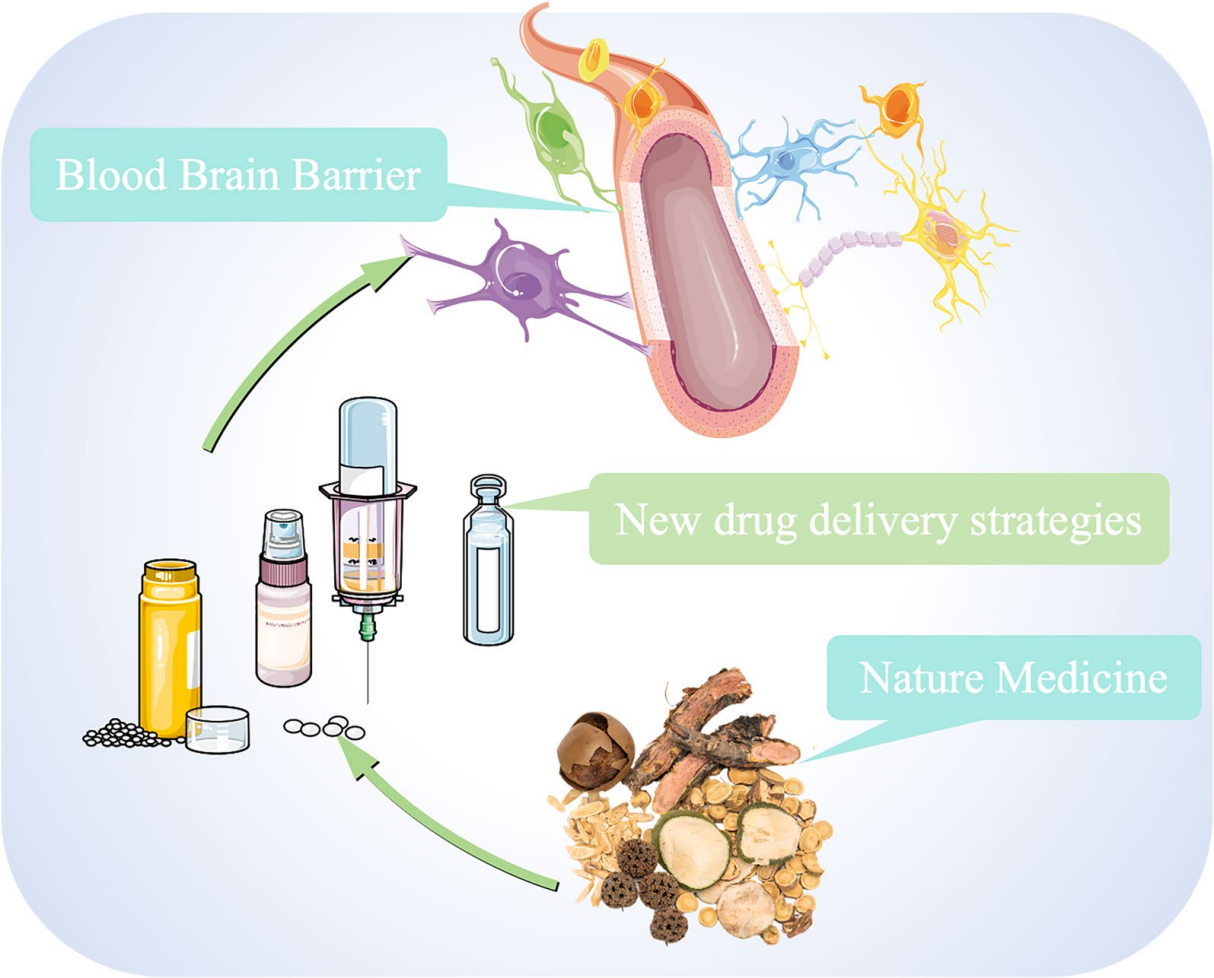
TCM and NDDSs for the treatment of depression. With the help of new drug delivery strategies, traditional natural medicines can overcome the blood‒brain barrier and to provide greater advantages in the treatment of depression

In natural medicinal chemistry research, the active ingredients found in TCMs primarily exert their efficacy through a population of small molecules, which includes oligosaccharides, saponins, terpenoids, and alkaloids, among others [43]. However, the in vivo delivery of small molecule drugs is largely contingent on their physicochemical properties, which significantly influence their bioavailability. Accordingly, efforts to improve drug solubility, control release, optimize activity, and adjust pharmacokinetic (PK) properties constitute the primary directions of research in the delivery of small molecule drugs [44]. Over time, scientists have explored new therapeutic functions by optimizing the therapeutically active components of new generations of drugs, gradually discovering multiple forms of drug intervention, such as proteins and peptides, monoclonal antibodies (mAbs), nucleic acids and living cells [45] (Fig. 2).

A number of highly active peptides have been extracted and isolated from Chinese medicines. These peptides have multiple antitumour, antioxidant, and immunomodulatory effects [46]. For example, the well-studied bitter melon MAP30 is a glycoprotein with a relative molecular mass of 30 kD, which specifically induces apoptosis in tumour cells, inhibits cancer cell proliferation, and prevents liver cancer [47] The results of antitumour animal experiments showed that the two-ring hexapeptide alizarin can extend the lifespan of mice with tumours, inhibit the incidence of tumours, and prevent the metastasis of tumour cells [48]. In addition, glutathione, which is present in many traditional Chinese medicines, including Ganoderma lucidum, Panax ginseng, Lycium barbarum, Gypenum chinense, Panax notoginseng, Polygonum multiflorum, Astragalus membranaceus, and Acanthopanax senticosus, and in foods such as malt, yeast, and shiitake mushroom, is effective in improving liver function, delaying ageing, and enhancing immunity [49]. Trichosanthin can enhance humoral immunity by changing the proportions of T cells with different functions [50].

The new functionality poses additional challenges, particularly in terms of stability (proteins and peptides), requirements for intracellular delivery (nucleic acids), and viability and expansion (living cells). To address these challenges, drug delivery strategies must evolve continuously [51]. For all drug therapies, the aim of drug delivery is to reach the target site in vivo by the transport or release (active or passive) of the pharmacophore, which in turn achieves drug efficacy maximization with minimized off-target effects [52]. This can be achieved by modulating drug PK properties, reducing drug toxicity, increasing target accumulation of drugs, and improving patient acceptance and adherence, and the development and innovation of drug delivery technologies are important strategies to further achieve this goal [53].

NDDSs represent a groundbreaking pharmaceutical technology that stems from the convergence of multidisciplinary theories and technologies in areas such as physicochemistry, biology, polymer science, material science, mechanical science, and electronics [54]. NDDSs have obvious advantages over traditional drug delivery methods [53]. First and foremost, NDDSs have the potential to enhance the stability of drug intervention effects over an extended period of time. This not only reduces the fluctuation of drug release rates but also mitigates the occurrence of peak and valley effects associated with fluctuations in blood drug concentration. Additionally, NDDSs can regulate the distribution of drugs in the body, achieving targeted pooling of drugs at the site of a lesion to reduce adverse effects and minimize the toxic side effects of the drug. Furthermore, NDDSs can adaptively adjust the dosage and administration frequency in consideration of biological rhythms to ultimately achieve optimal therapeutic effects [55].

## Novel drug delivery carriers

**Fig. 3:**
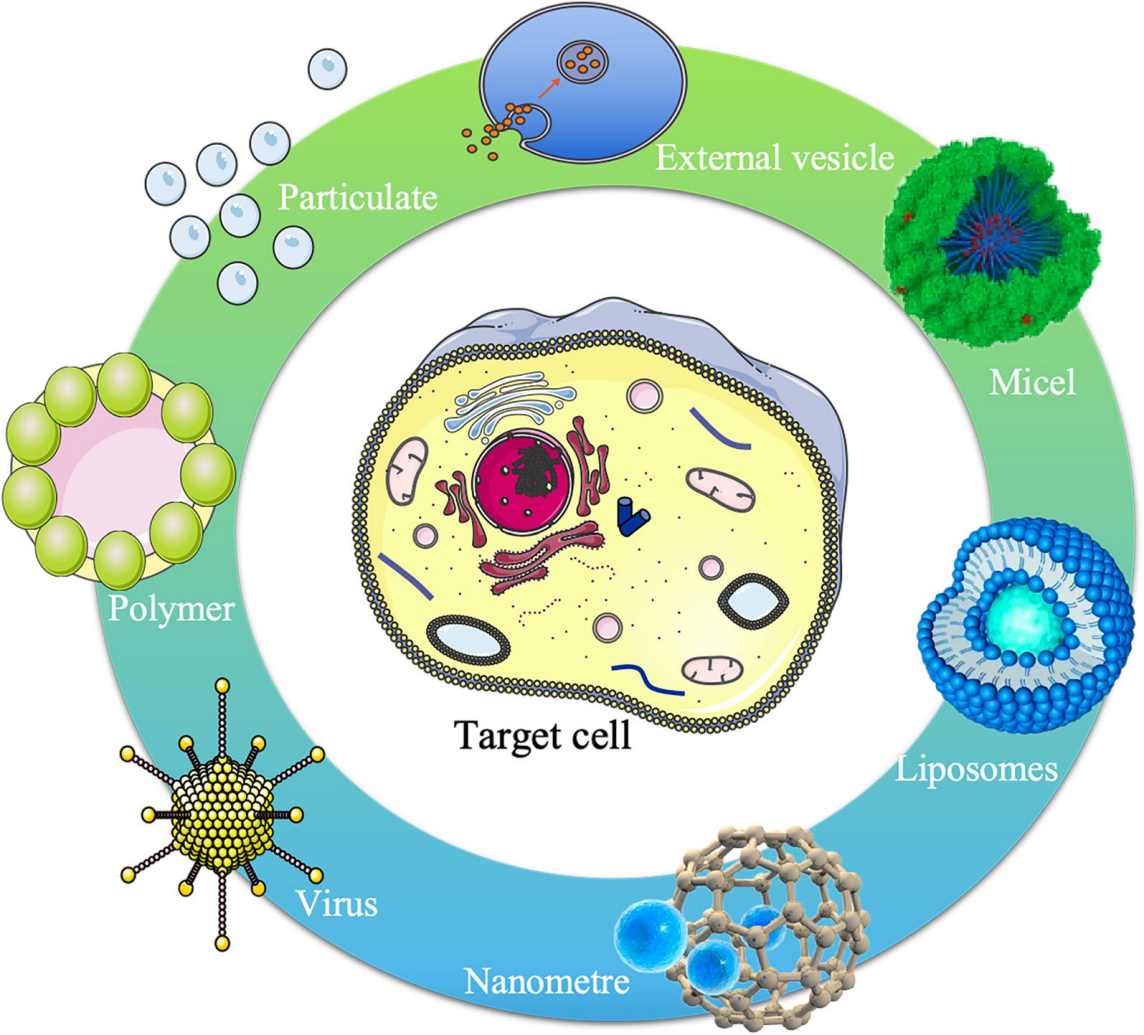
Novel drug delivery carriers. Research on drug delivery carriers, including nanocarriers, liposome carriers, prodrug carriers, targeted formulation delivery carriers, virus carriers, and exosome carriers, is currently the main direction for the development of novel drug delivery carriers

The advancement of novel drug delivery vehicles is essential to enhance drug bioavailability and exert pharmacodynamic effects. In addition to factors of biocompatibility, the study of novel drug delivery carriers should also take into account the physicochemical characteristics of drugs and their compatibility with the design and intended use of the carrier [56] (Fig. 3). The current focus in the development of drug carriers is on overcoming physiological and pathological barriers within the organism [57]. This includes enhancing the concentration of active molecules near the target, improving the pharmacokinetic properties of active molecules, and regulating the controlled release and degradation of metabolites [58].

Currently, oral administration is considered the most convenient and highly adherent mode of drug delivery [59]. To improve the dosing efficiency, a fundamental strategy is to enhance the gastrointestinal biological barrier capability of drug carriers when administered orally. This can be achieved by protecting the drug from the harsh degradation environment of the gastrointestinal tract and increasing the residence time of the targeting moiety. Other approaches may include increasing bioadhesion to enhance drug absorption or circumventing the limitations found in conventional dosage forms to improve the efficacy of drugs [60].

The bioavailability of the active ingredient is a crucial factor in drug development, and although certain biomolecules possess intervening properties in organisms, their application is often limited by low bioavailability [61]. Low bioavailability is typically attributed to low water solubility or low membrane permeability. Given that the human body is made up of 65% water, drugs must exhibit some hydrophilicity or polarity for solubility in water and subsequent functionality. Simultaneously, due to the structural composition characteristics of the cell membrane, drugs must also display lipophilicity or nonpolarity to pass through the lipophilic cell membrane [62]. With developments in pharmaceutical science, an increasing number of drugs are made up of biomolecules, such as peptides, proteins, oligosaccharides, and DNA. These molecules often present low bioavailability characteristics that need to be protected against catalytic degradation by enzymes and acids [63]. Common drug carrier systems include liposomes and vesicles composed of phospholipids and nonionic lipids combined with surfactants, polymeric micelles formed by charged or neutral block copolymers, and nano- and microparticle carriers created through various processes [64] (Table 2).

**Table 2:** Novel drug delivery carrier

Type of drug delivery vehicle	Drug delivery vehicle characteristics	References
Nanoparticles	Advantages of small size, a large specific surface area, controllable morphology and easy modification Can effectively embed, adsorb or covalently crosslink hydrophilic/hydrophobic drug molecules Enhance the local concentration of drugs in the body	[66, 67]
Liposomes	Are able to bind to a wide range of biologically active compounds when used as a drug delivery vehicle due to their inherent amphiphilic nature	[68, 69]
Polymer	Encapsulate the drug in a specific part of the polymer Carry drugs by means of polymer-drug conjugation Employ targeting factors to load drugs for targeting to specific disease sites	[70, 71]
Prodrugs	Produce synergistic effect Expand the scope of clinical applications Improve drug absorption Improved blood drug concentration drug efficacy is a function of drug concentration at the site of action Slow release at the administration site to achieve a prolonged action time	[73, 74]
Targeting drug delivery system	Gives the drug a specific pharmacological activity Increases drug targeting ability and retention in target tissues Reduces drug toxicity to normal cells Improves the bioavailability of pharmaceutical formulations	[76, 77]
Virus	High safety Low toxicity Contains marker genes	[78, 79]

### Nanobased drug delivery vehicles

Nanotechnology formulations have the potential to substantially enhance the solubility and stability of active ingredients found in Chinese medicines. This technology facilitates cellular uptake and efficacy by improving targeting to pathological sites, thereby overcoming the present challenges faced in the application of active ingredients found in TCMs, including a sluggish onset of action, low utilization rate, cumbersome processing, and high dosage requirements [80]. Common nanocarrier structures include polymers, nanoparticles, nanoemulsions, nanomicelles, etc.

Polymeric drug delivery systems share similarities with liposomes and possess physical and chemical properties such as biodegradability, biocompatibility, water solubility, and storage stability [81]. They are considered ideal drug delivery materials. Based on the structural characteristics of the polymer, these systems can be categorized into two forms: those that encapsulate the drug in a reservoir within the polymer coating (reservoir type) and those that embed the drug into the polymer matrix (monolithic type). Drugs can also be carried in a polymer-drug conjugate manner, and targeting factors can be introduced to transport drugs to specific disease sites through the physical and chemical properties of the polymer, such as active or passive targeting [82]. When polymer-based chemotherapy drugs are bound to water-soluble macromolecules, the resulting drug exhibits improved penetration into brain tissue due to the prolonged retention time of the drug [83].

Nanoparticles can be created for drug adsorption, attachment, or encapsulation within carrier materials [84]. Surface modifications, such as ligands or antibodies, can actively target corresponding receptors, antigens, and the like in vivo, thus facilitating drug uptake [85]. Duan Xiaoying et al. employed polyethylene glycol-poly(lactic acid-hydroxyacetic acid) (PEG-PLGA) block copolymers as nanocarriers, achieving an entrapment efficiency of up to 90.05% for matrine nanoparticles sized 112.04 nm. This strategy significantly enhanced the storage stability of matrine [86].

A nanoemulsion refers to a homogeneous dispersion of water, oil, surfactant and cosurfactant in appropriate proportions to ensure low viscosity, thermodynamically stability, and either transparency or translucency [87]. The use of nanoemulsions has the ability to extend the action time of drugs in the body and significantly enhance the bioavailability of herbs within the body [88]. Kotta et al. utilized response surface methodology to optimize the formulation and process of a resveratrol nanoemulsion. In addition, they evaluated the in vitro drug release, in vitro permeation, and brain targeting properties of the nanoemulsion. Their findings demonstrated that the in vitro nasal mucosal permeation rate of this system was approximately twice that of the active pharmaceutical ingredient (API). Furthermore, brain-targeting studies revealed higher concentrations of resveratrol in the brain when administered as a nanoemulsion than as a suspension, proving that this dosage form is more effective in improving the bioavailability of herbal medicines [89].

Nano-micelles are amphiphilic block copolymers that self-assemble in water through the combination of hydrophilic and hydrophobic blocks. The hydrophobic segments of these copolymers aggregate into a hydrophobic core under the influence of water molecules, while the hydrophilic segments form a stable hydrophilic outer layer within the water [90]. Micelles are both easy to prepare and suitable for large-scale batch production, making them ideal for a variety of applications. They offer enhanced drug solubility, good biodegradability, small particle size, high stability, and great functionality, making them an excellent choice for brain-targeting nanocarriers. The use of chemical polymer materials also helps to minimize risks, such as microbial contamination and immune resistance [91]. In 2021, paclitaxel micelles were approved as a new class 2 drug in China. This improved dosage form was created using an innovative pharmaceutical excipient: an amphiphilic block copolymer of methoxy polyethylene glycol-poly (propylene glycol) (mPEG-PDLLA 53/47) as a carrier. These micelles were approved in combination with cisplatin for the first-line treatment of patients with epidermal growth factor receptor (EGFR) mutation-negative and mesenchymal lymphoma kinase (ALK)-negative, nonsurgically resectable locally advanced or metastatic non-small cell lung cancer (NSCLC), presenting certain safety and efficacy advantages [92].

### Liposome-based drug delivery vehicles

The concept of liposomes was originally introduced by Bangham et al. in 1965. Today, it commonly refers to small closed vesicles with a bilayer wrapped in an aqueous structure formed by dispersing phospholipids and other lipids in water. Due to their structural similarity to biological membranes, they are also known as artificial biological membranes [93]. As lipid-based drug carriers, liposomes are of great interest and have been extensively studied. Not only are they made from phospholipids, an intrinsic component of human cells, making them biocompatible and nonimmunogenic, but they can also be prepared as nanoscale particles, facilitating the penetration of biological barriers such as blood vessel walls and cell membranes [94].

It has been shown that lipid-soluble molecules (molecular weight < 500 Da) can cross the BBB through the small pores formed transiently within the lipid bilayer [93]. Drug delivery across the BBB can be improved by incorporating hydrophobic groups into the molecule and promoting passive diffusion [95]. Yan Dekang et al. investigated the in vitro targeting ability by optimizing the prescription of mannose-modified curcumin/ginsenoside Rb1 coloaded liposomes. The final prescription preparation of MAN-Cur/GS-Rb1-Lips was successfully optimized, resulting in significantly improved brain targeting ability. These MAN-modified Cur/GS-Rb1-Lips are considered promising brain-targeting nanodelivery systems [96].

### Prodrug-based drug delivery vehicles

Carrier prodrugs are compounds composed of active drugs and carriers that exhibit little or no activity in vitro. These carriers release active drugs through enzymatic or nonenzymatic conversion in vivo [97]. Precursor drug carriers are typically less active than the parent compound [98]. The carrier structure, typically lipophilic, must be biocompatible and capable of releasing the active compound as needed [99]. For instance, prodrug carriers are frequently utilized to enhance the bioavailability of oral penicillin drugs [100].

A properly designed prodrug carrier should satisfy several requirements, including the following: the prodrug should be inactive or less active than the parent drug; typically, the drug is covalently bonded to the carrier, and the bond between the drug and carrier must be broken in vivo; both the prodrug and the carrier released in the body must be nontoxic; and to ensure that an effective concentration is attained at the target site and to reduce direct metabolism or gradual inactivation of the prodrug, the release of the parent drug should be sufficiently rapid [101]. The synthesis of prodrug carriers generally involves utilizing the polar functional groups present in active compounds and drug molecules [102]. By means of this approach, prodrugs are synthesized through the amidation or esterification of the amino, carboxyl, and hydroxyl groups within the parent drug; this increases their lipid solubility and enhances their efficiency for brain uptake. As these groups are subsequently hydrolysed, the release of active drugs into the brain is facilitated [103]. For drugs that contain alcohol or carboxylic acid groups, ester is the most commonly used prodrug form. Amines, on the other hand, can be transformed into amides, imines, azo, amine methylation, or other forms to prepare prodrugs. Carbonyl-containing drugs can be prepared using a Schiff base, oxime, acetal, or ketal, among other methods. Jing Lanlan et al. investigated the design and synthesis of the A11 prodrug Chuanxiongzine, an anti-ischaemic stroke drug, and discovered that it exhibits significant permeability across the BBB [104].

### Targeted formulation-based drug delivery vehicles

A targeted drug delivery system is a formulation that effectively targets the drug to the desired site of action (target area) and demonstrates minimal or no interaction with nontarget tissues [105]. The notion of targeted agents was first introduced in Ehrlich. Nevertheless, due to the prolonged limitations in human understanding of diseases, the failure to comprehend drug action at the cellular and molecular levels, and obstacles in materials for and the preparation of targeted agents, challenges have been encountered in the use of targeted agents [106]. Only with remarkable advancements in molecular biology, cell biology and materials science have new opportunities emerged for the evolution of targeted agents [107]. In the current era, scientists are vigorously and comprehensively examining targeted agents, including their preparation, properties, in vivo distribution, targeting, and pharmacological and toxicological effects.

Physical and chemical targeting preparation involves the utilization of physicochemical methods to create a targeted preparation capable of exerting pharmacodynamic effects at a precise location [108]. A popular approach entails the use of a temperature-sensitive carrier to produce a thermosensitive formulation, which, when exposed to local hyperthermia, enables controlled drug release at the designated target site [109]. Moreover, it is feasible to employ pH-sensitive vehicles to prepare pH-sensitive formulations that facilitate drug release exclusively within specific pH target regions [110].

Magnetic targeting is a directional method that utilizes a magnetic field to guide the movement of drug-loaded magnetic microparticles to the site of a lesion [111]. By integrating magnetic substances into drugs, a magnetically targeted formulation may be created that can be directed through blood vessels to precisely localize at a targeted area via an extracorporeal magnetic field [112]. Yan Runmin and colleagues developed a complex particle composed of magnetic paclitaxel-iron tetroxide-drug-loaded liposomes that can be directed to the intercellular matrix of brain tissue and penetrate cells through the BBB by means of magnetic field guidance in vitro. This novel strategy effectively enhances the concentration of chemotherapeutic drugs at the target site and greatly improves antitumour efficacy [113].

pH targeting is a theoretical principle that utilizes the distinct pH characteristics of pathological tissues, cells, or specific cellular regions during disease states to select appropriate materials [114]. Conghui Chen and colleagues leveraged the pH-sensitive properties of the target region to develop a novel lipomimetic paclitaxel-loaded nanocarrier (bsa-lc/dope-ptx) with targeting and pH-sensitive capabilities [115].

### Virus-based drug delivery vehicles

The primary purpose of viral vectors was initially to address the challenge of delivering DNA, and many researchers have utilized them for this purpose in the treatment of genetic disorders [116]. The earliest successful treatment case was observed in a long-term clinical trial under the guidance of Dr. William French Anderson at the National Institutes of Health in the United States. In this trial, a retrovirus was used to transport normal adenosine deaminase (ADA) genes to T cells isolated from patients, which were then reinfused into the patient’s body to treat children with ADA-deficient severe combined immunodeficiency disease (ADA-SCID) [117].

A viral vector involves the application of genetic engineering techniques to convert a virus into a delivery vector for foreign genes. It infects cells, transfers foreign genes into cells, and enables long-term gene expression [118]. Viral vectors possess numerous benefits, such as high transfection efficiency and high levels of exogenous gene expression, and are widely applied in areas such as basic research, gene therapy, and vaccine development [119]. The three major types of viral vectors include retroviruses (RVs), lentiviruses (LVs), and adenoviruses (ADVs) [120]. During the Novel Coronavirus Pneumonia Epidemic, the Novel Coronavirus Pneumonia vaccine was developed using a viral vector approach. Adenoviral DNA was extracted using various cell lysis agents and detergents, which helped break down the cell membrane and extract nonnucleic cellular material. Subsequently, after whole-genome sequencing, the E3 region of the adenovirus genome was removed to make room for Severe Acute Respiratory Syndrome Coronavirus 2(SARS-CoV-2) spike protein integration [121]. Apart from this, some research studies have isolated the cDNA of cytotoxic proteins from traditional Chinese medicines, which were then used to transport these genes to malignant cells via a recombinant adeno-associated virus. The results demonstrated that the toxic protein-encoding gene of the traditional Chinese medicines had a significant inhibitory effect on malignant cells [122].

### External vesicle-based drug delivery vehicles

Extracellular vesicles (EVs) are small vesicles released by cells that contain biologically active molecules such as proteins and miRNAs [123]. In 2013, the Nobel Prize in Physiology or Medicine was awarded to American scientists James E. Rothman and Randy W. Schekman and German-American scientist Thomas C. Sudhof for their discovery of the transport regulatory mechanism of extracellular vehicles (EVs) [124]. EVs have been recognized as the most promising drug delivery vehicles due to their natural material transport properties, inherent long-term circulation ability, and excellent biocompatibility, and they are suitable for delivering various chemicals, proteins, nucleic acids, and gene therapy agents. Additionally, EV drug delivery have the advantage of being able to cross the blood‒brain barrier [125]. EVs can be classified based on their biogenesis or release pathway, including exosomes, microvesicles/microparticles, apoptotic bodies/blebs, large oncosomes, and other EV subsets. The EV membrane can resist the degradation effect of extracellular nucleases, making it a suitable carrier for small nucleic acid drugs [126, 127]. Currently, there are three main types of EVs used for vector development in industry: human engineered exosomes, exosomes derived from human mesenchymal stem cells (MSCs), and exosomes derived from red blood cells/platelets and other nonnucleated cells [128, 129]. Engineered exosomes are currently mainstream in industry because their targeting and drug loading efficiency can be enhanced [130]. MSC-derived exosomes are approximately 40–80 nm in size and can deliver 20–30 bp small RNAs, while red blood cell-derived exosomes can carry up to 30 kb of DNA due to the characteristics of red blood cells [131].

## Novel routes of drug delivery

**Fig. 4:**
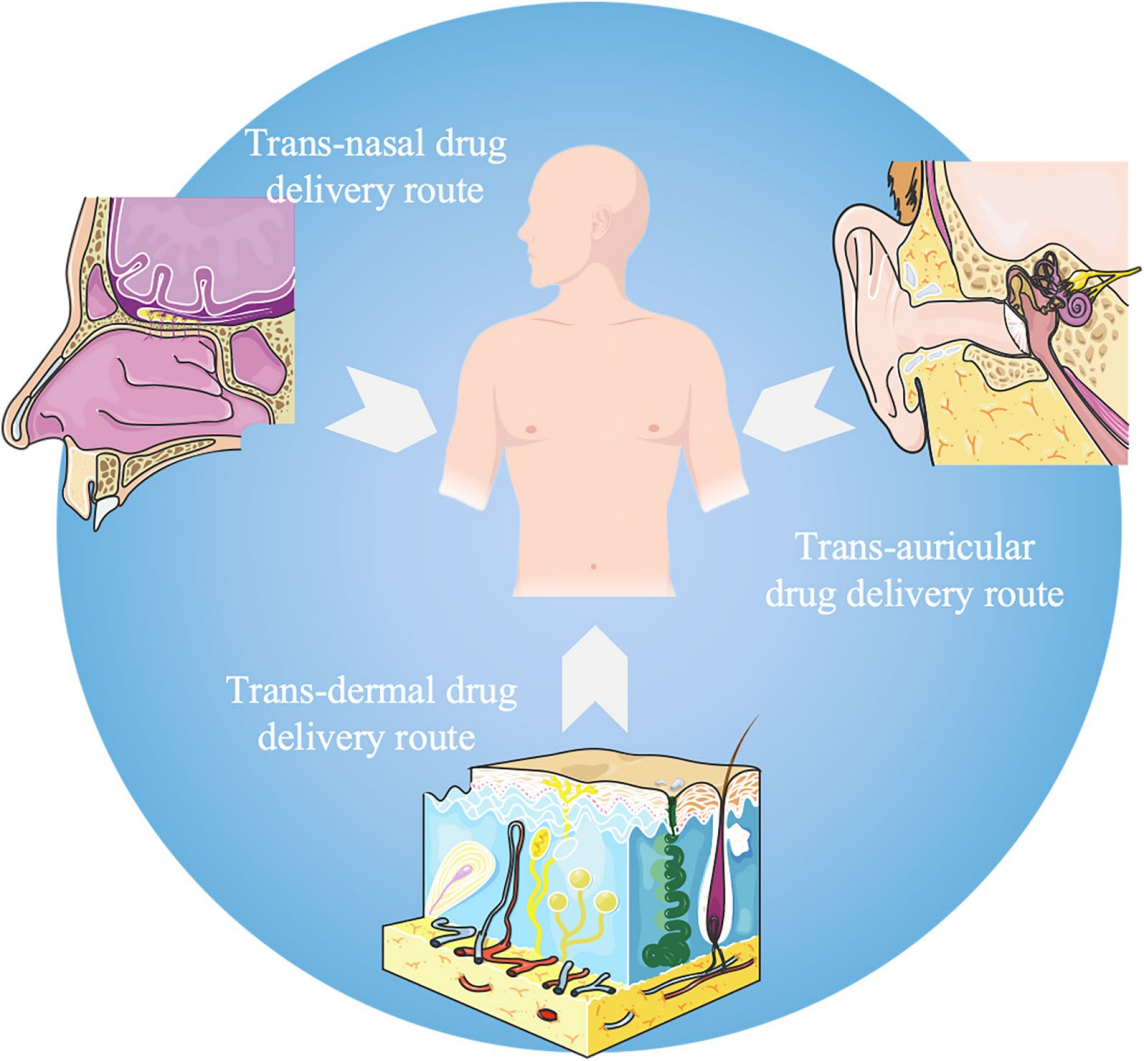
Novel routes of drug delivery. The new routes of drug delivery for antidepressants currently under investigation include the following recent directions: the transdermal route, the intranasal route, and the transmural route

The therapeutic effect of a clinical condition is closely correlated with the administration route of a drug, and the efficacy of the same drug may vary greatly depending on the method of administration (Table 3) [132]. Advancements in science and technology have resulted in drug dosage forms that offer greater convenience in clinical treatment. When selecting medications, doctors must consider the characteristics of the drug and the desired treatment outcome before determining the appropriate form [133]. The exploration of novel administration routes represents an innovative approach to drug delivery that builds upon traditional methods, thereby potentially enhancing drug efficacy (Fig. 4).

**Table 3:** Novel route of administration

Route of administration type	Route of administration characteristics	References
Transdermal administration	Means that a drug is absorbed by the skin into the systemic blood circulation and achieves an effective blood concentration, achieving the purpose of clinical disease treatment or prevention	[134–136]
Nasal administration	Refers to a class of preparations administered through the nasal cavity to exert local or systemic therapeutic or preventive effects Especially suitable for those drugs that are difficult to administer except by injection and need to play a systemic role Similar to oral administration, difficult to absorb polar drugs Drugs unstable in the gastrointestinal tract Drugs and proteins and peptides with strong hepatic first-pass effects	[137–139]
Ototopical administration	Ear topical administration has many advantages, the most significant advantage is to be able to obtain a higher local drug concentration than systemic administration The main limitation of topical administration is the potential ototoxicity of certain drugs, especially when the drug concentration is very high	[140, 141]

**Fig. 5:**
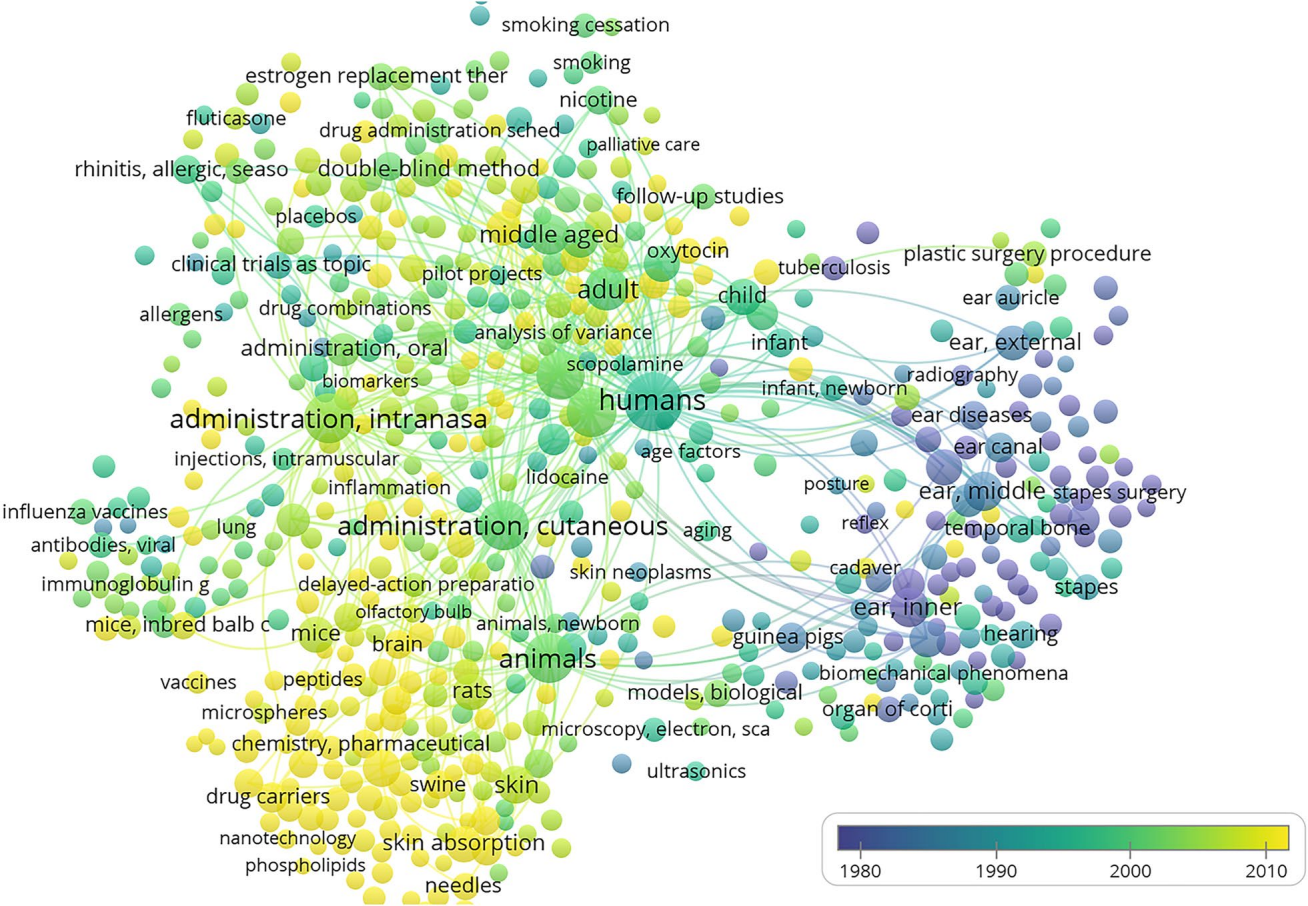
Visual analysis of novel drug delivery routes. Analyzing the co-occurrence frequency of keywords in the literature data related to new drug delivery routes using a bubble chart. The size of the bubbles represents the frequency of keywords, and the thickness of the links between bubbles represents the degree of connection between keywords. The results show that the transdermal route, intranasal route, transcutaneous route and other drug delivery routes are currently the main research targets of new drug delivery routes

Based on this, the bibliometric statistical method was used in this study; the CBM, Wanfang Data, Pubmed, Web of Science, Cochrane, CNKI, and Vip Information Chinese journal service platform were selected as the search databases; and the research trends of novel routes of administration were analysed with “Novel route of administration” as the search term. The search yielded 26,973 journal articles related to the “Novel route of administration” since the library was built. All retrieved articles were imported into an EndNote database, 7963 duplicate articles were deleted. The remaining 20,010 articles were imported into VosViewer for the analysis of keyword co-occurrence frequency (Fig. 5). The analysis revealed that current routes of administration, such as transdermal administration, nasal administration, and ototopical administration, were the main research subjects to discuss novel routes of administration.

### Transdermal administration

Transdermal drug delivery systems can be utilized as an effective means of medication administration to assist with drug application when patients experience intolerable gastrointestinal side effects or swallowing difficulties associated with traditional oral dosage forms [142]. The transdermal method of drug administration not only extends the drug's duration of action but also aids in avoiding negative reactions. Furthermore, transdermal drug delivery provides the benefits of maintaining steady blood concentrations, increasing the drug's duration of action, decreasing fluctuation in drug metabolism among different patients and even within an individual patient, bypassing first-pass metabolism, and potentially attaining lasting and foreseeable therapeutic results [143]. As a noninvasive method of drug delivery, transdermal administration may be particularly well suited for patients who have difficulty taking medication independently or those who experience cognitive impairment [144].

For example, in nicotine withdrawal therapy, a nicotine agonist is formulated into patches that are applied to the skin to aid smoking cessation [145]. Studies have shown that these patches can effectively alleviate symptoms of cognitive impairment associated with neurodegenerative disorders, psychosis, schizophrenia, attention disorders and more [146]. studies have confirmed that the neuroprotective properties of nicotine derive from its ability to stimulate A7 nicotinic receptors. When A7 nicotinic receptors are activated, they increase the release of striatal dopamine. This, in turn, protects the nigrostriatal pathway and helps to preserve neural tissue [147]. Ganoderma lucidum is known for its ability to activate blood circulation and remove blood stasis, making it an effective treatment for stroke and hemiplegia. One of the key active ingredients in Ganoderma lucidum is triterpenes. Recent research has shown that combining Ganoderma triterpenes with a nanosuspension gel can significantly enhance skin retention of the drug and its local bioavailability, making it an effective treatment option [148].

In regard to transdermal drug delivery, there are a few different methods that can be employed. Along with drug patches, percutaneous penetration therapy can also be utilized. Subcutaneous implantation may prove to be beneficial in the treatment of certain mental disorders. Research has shown that haloperidol biodegradable polylactic-coglycolic acid (PLGA) pellets implanted via surgery can provide steady drug release for up to 5 months in vitro. This approach has proven to be a more effective means of treating long-term psychotic episodes than traditional drugs. Furthermore, in vivo animal models have demonstrated increased expression of striatal dopamine 2 (D2) receptors, validating the effectiveness of this drug delivery system [149]. Rabin et al. conducted a study using risperidone PLGA implanted micropills and found a sustained drug release pattern lasting at least 2 months. These results confirm the efficacy of this delivery system for the long-term treatment of schizophrenia [150]. Compared to traditional dosage forms, subcutaneous implantable dosage forms have the advantage of reducing hepatic metabolism and decreasing peripheral drug inactivation, as well as possessing higher bioavailability. Additionally, the long-term drug release characteristics can provide patients with sustained relief and stability.

### Nasal administration

The BBB poses a significant obstacle to the transport of various therapeutic agents to the brain, and as of yet, we have been unable to administer most drug molecules in effective therapeutic doses. To overcome the hindrances of the BBB, researchers have begun exploring novel pathways for drug delivery, such as the nasal-brain transport approach [151]. The intranasal drug delivery route offers the unique advantage of delivering drugs directly to the brain, thereby enhancing site specificity within the brain while avoiding systemic side effects. Moreover, this approach has been shown not to cause any detrimental effects on the nasal mucosa or the central nervous system [152]. Although the complex nasal structure, mucociliary clearance, enzymatic degradation within the nasal cavity, and pathological conditions such as rhinitis and the common cold are the main controversies in nasal drug delivery, current nanotechnology methods using solid lipid nanoparticles, polymer nanoparticles, nanoemulsions, liposomes, and polymer micelles provide the ability to overcome these barriers [153].

According to Chinese medicine theory, “the nose serves as a direct connection between the brain and the external environment. The brain plays a regulatory role over the nose, while the nose’s function relies on the brain, The Governor Vessel meridian intersects at the nose, and the nasal orifice is linked to the brain through both the Governor Vessel and the bladder meridian of the sun” [154]. The theory regarding the connection between the nose and the brain has been extensively covered in numerous ancient Chinese medicine texts. For instance, both the *'Complete Book of Sores and Ulcers'* and the *'Miraculous Pivot'*, which are regarded as traditional Chinese medicine classics, have detailed the intricate relationship between the nose and the brain [155]. Numerous applications of traditional Chinese medicine theory in treating patients have been documented over time. One such example can be found in the *'General Medical Collection of Royal Benevolence'*, a classical text devoted to traditional Chinese medicine that records the treatment of migraines through the use of a nasal drip made from Chinese radish seed and ginger juice. Another ancient book of traditional Chinese medicine, the '*Prescriptions for Universal Relief*', recommends the application of garlic juice directly into the nose after its skin has been removed and ground into juice for treating headaches. Finally, in the '*Prescriptions for Emergent Reference*', another classical text of traditional Chinese medicine, raw radish juice is injected into the nose to help mitigate migraines [156].

A team led by Ju Aichun found that intranasal administration of salvianolic acid B significantly increased the number of newly formed neurons in the hippocampus of rats with cerebral ischaemia, resulting in a proliferation of neuronal cells and an overall protective effect on these cells. This ultimately led to improved learning and memory abilities in the rats that had suffered cerebral ischaemic injury [157]. In addition, Chinese herbal extracts, such as curcumin and ligustrazine, packaged in nanoparticles have been shown to effectively improve ischaemia‒reperfusion injury in animal models when administered via the intranasal route [157]. A study conducted by Sun Yaping et al. showed that the short-term intranasal administration of genistein (Gen) led to a significant reduction in cerebral infarct size, improved behavioural function, and decreased neuronal damage [158]. The intranasal administration of Panax ginseng saponin was found to significantly alleviate cerebral oedema and stroke symptoms resulting from cerebral ischaemia‒reperfusion in gerbils, while administering the same dose of an oral solution had minimal effect [159].

### Ototopical administration

The *'Inner Canon of the Yellow Emperor'* (the most authoritative text of early medical theory and drug therapy), a classical text on Chinese medicine, associates the ear with the brain and suggests that damage to the brain can result in damage to the ear [160]. Furthermore, the Chinese medical text ‘*Suwen-Xuanji-original disease Formula*’ contains records of acupuncture and magnetic therapy being used to treat deafness. The traditional Chinese medicine approach to treating ear issues is a distinct and specialized technique within this field [161]. Based on Chinese medicine principles, the ear is interconnected with internal organs and meridians. Consequently, when medication is administered via the external ear canal, it can travel through the meridian and access the internal organs, successfully treating illnesses and even saving lives [162, 163]. Medicated earplugs are capable of treating not only ear-related conditions but also various systemic ailments. For instance, the Chinese medical text ‘*Hui Sheng* Ji’ notes that when treating blindness, “small centipeda herb should be applied by blowing the nose, plugging the ears, and pasting it on the eyes”. Such treatment is believed to possess remarkable healing properties. [164].

The selection of an appropriate route for ear medication is contingent upon the nature of the disease and the physicochemical properties of the drug in question. Typically, antibiotics and antifungal drugs are administered via topical drops and gels [165]. Local administration of drugs to the ear has many advantages, the most significant of which is the ability to achieve higher local drug concentrations than systemic administration. However, the main limitation of local administration is the potential for some drugs to cause ototoxicity, particularly at very high drug concentrations, which can lead to significant damage to the auditory organs [166]. Ear administration primarily involves three pathways: (1) Transtympanic drug delivery. The tympanic membrane serves as the first barrier for delivering drugs to the middle and inner ear; hence, the challenge with administering drugs through the tympanic membrane is ensuring that the drug penetrates the intact membrane and then diffuses through the ear canal into the middle and inner ear. One way to facilitate this process is through the use of permeation enhancers; (2) transtympanic administration, which begins with the injection of a drug into the middle ear cavity. The drug then diffuses into the cochlea. The diffusion rate depends on factors such as molecular weight, conformation, concentration gradient, lipophilicity, charge, and the thickness of the circular window film; and (3) intracochlear drug delivery, which bypasses the middle ear and allows direct delivery of the drug to the intended site, yields better bioavailability of the substance compared to other routes [167].

Chen Gang's research team has implemented PLGA nanoparticles as carriers to integrate contemporary medical theory and novel formulation technology into the study of Chinese medicine compound components, resulting in the development of a new model of Chinese medicine compound inner ear drug delivery systems. The results of their research demonstrate that PLGA nanocarriers effectively deliver the multicomponent Chinese herbal formula to the inner ear and brain, leading to improved local bioavailability. Additionally, the biphasic drug release system based on PLGA nanoparticles not only enhances the local bioavailability of the multicomponent Chinese medicine compound across the round window membrane but also prolongs the retention time of the drug in vivo. Furthermore, pharmacodynamic and preliminary toxicity evaluations have validated that the inner ear nanodelivery system of Salvia miltiorrhiza is efficacious in treating age-related deafness or cerebral ischaemia without causing any functional damage to the inner ear [168].

## Novel drug delivery systems in Chinese medicine research

**Fig. 6:**
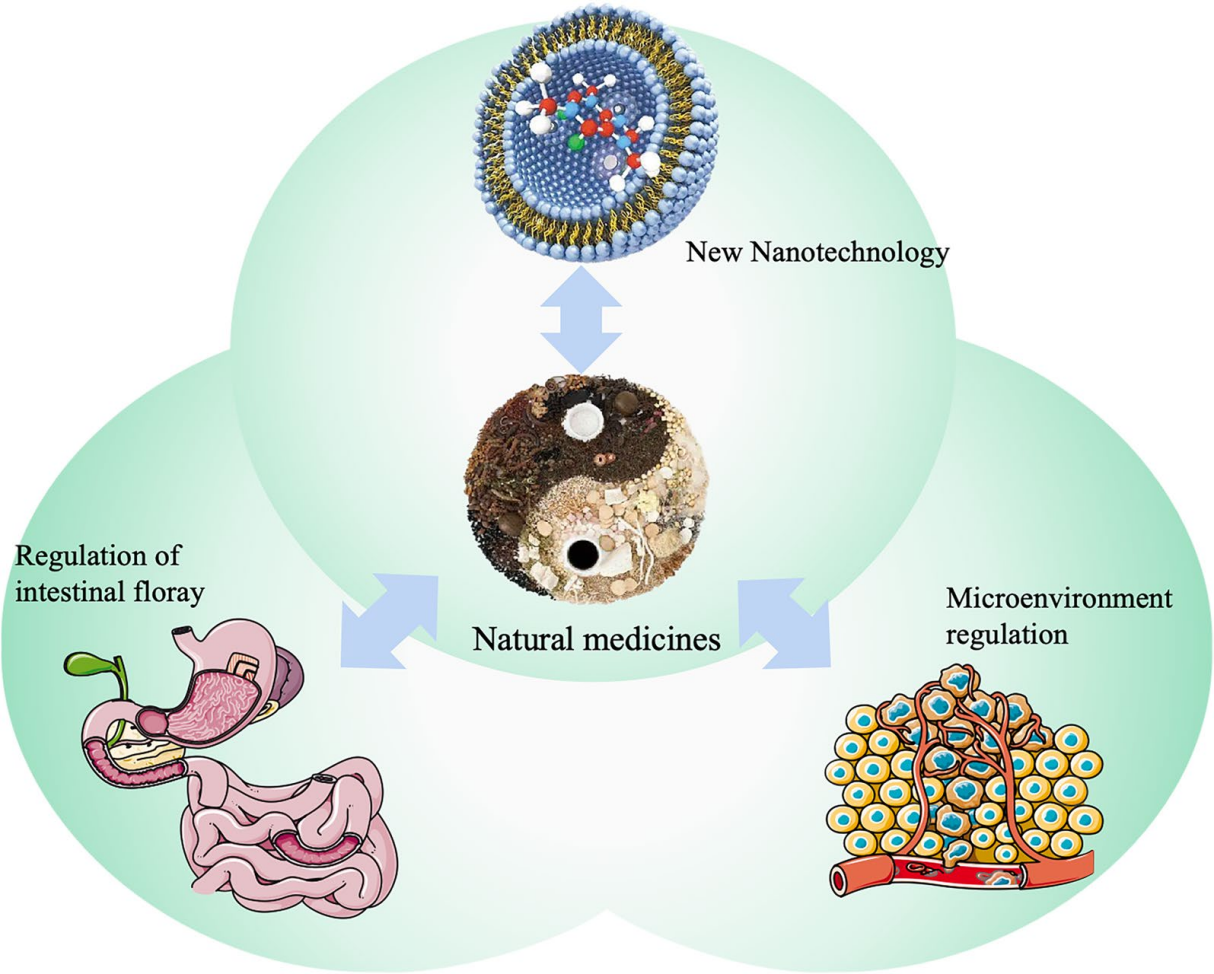
Novel drug delivery systems for Chinese medicine applications. In the field of research on interventions for nervous system diseases using natural medicines, the development and application of new drug delivery technologies can lead to further progress in research avenues such as nanodrug delivery methods, microenvironment modulation methods, and gut microbiota modulation methods

The NDDS industry for TCM formulations has been thriving thanks to technological advancements. The application of TCM alternative therapies has evolved with the development of a new drug delivery system [169]. TCM originated in ancient China and has been widely used in Asian countries for over two thousand years, and it currently plays an important role in the treatment of neurological diseases with the help of new drug delivery systems [170] (Fig. 6).

Currently, significant obstacles are being faced in the development of novel drug delivery systems in traditional Chinese medicine (TCM) research. These include the complex composition of compound formulations, the challenging identification of quality control indicators, and the difficulty in establishing pharmacokinetic methods in vivo [171]. In future development, we must undertake basic research on drug analysis, quality control, and in vivo pharmacokinetics. Additionally, we need to independently develop new drug delivery systems that align with current trends in this area. It is also critical to incorporate new drug delivery systems from both domestic and international sources in the development of traditional Chinese medicine and natural medicine in a timely manner [172].

To achieve optimal drug delivery strategies and improve the efficacy of Chinese medicines, researchers have been actively pursuing in-depth research in three key areas: drug modification techniques, microenvironmental modulation methods, and drug delivery systems. The aim is to fully leverage the active properties of the molecule within a specific therapeutic window while also precisely controlling the release of the drug to extend its metabolic lifespan and maximize the therapeutic effect during this window (Table 4) [64].

**Table 4:** Novel drug delivery systems for Chinese medicine applications

Route of administration type	Route of administration characteristics	References
New nanotechnology	Highly effective in increasing the solubility and dissolution rate of insoluble drugs Reduced volume of drug delivery, reduced toxic effects Nanomedicines can also impart new properties to drugs to improve bioavailability and clinical efficacy	[173, 174]
Regulation of intestinal flora	Regulates the metabolism of the intestinal flora by modulating the composition of the intestinal flora and/or by regulating the activity of the enzymes that catalyse the production of metabolites This in turn leads to an increase or decrease in the content of certain metabolites, as well as the production of new metabolites or the disappearance of certain metabolites	[175]
Microenvironmental regulation methods	Provide new capabilities for various therapies by improving the stability of drugs in physiological fluids, controlling drug‒drug interactions in individual cells, and altering drug-target interactions Drug modifications have been able to address the changing structural complexity, specificity and function of various formulations and are now being used in the further development of cellular therapies	[176, 177]

Scientists have used drug modification to adjust the release rate of drugs to an ideal constant rate of release. For example, some scholars have combined two types of preservatives, including one that is hydrolyzable, as a template to prepare pH-sensitive mesoporous silica-based nanocontainers with extremely high effective payloads to achieve controllable rates of drug release [178]. Alternatively, controlled drug carriers can be injected or implanted into the body, and a remote-control system can be used by health care professionals to achieve precise and adjustable release rates in a “programmable on-demand” manner [179]. Maintaining a constant release rate is crucial for long-term drug administration, which is typically achieved through implanted devices. Important considerations for implanted drug delivery systems include the prevention of drug leakage, the size of the implant device, and methods for removal once the device has reached the end of its use. Collaboration between multiple disciplines, such as bioengineering and biomaterials, is required to address these challenges, and scientists have explored various possible approaches. For example, microfluidic drug delivery systems that offer both consistent release rates and adaptable responses to environmental conditions may enable high precision and predictability in drug delivery [180]. Nanoparticles composed of alginate have become one of the most widely used biomaterials for drug delivery and targeted delivery. Their advantages include their multifunctional physicochemical properties that allow for chemical modification and site-specific targeting as well as their biocompatibility, biodegradability, and mucoadhesive properties [181].

### New nanotechnology

The development of new drug delivery systems is a key focus of research and development efforts in the market. In particular, nanomedicine has emerged as a critical player in this field, given its proven efficacy in targeting specific organisms. By allowing more targeted delivery, nanomedicines address the issues associated with traditional drug delivery methods, such as the wide distribution of drugs throughout the body. This approach increases the specific affinity of the active molecule to the lesion, thereby avoiding nonspecific toxicity and other adverse side effects associated with high doses of the drug. As such, nanomedicine is viewed as a highly effective means of addressing the challenges involved in drug delivery [182]. The current progress in nanotechnology provides opportunities for overcoming limitations in the treatment of central nervous system disorders, and nanomedicine delivery systems are expected to increase the bioavailability of drugs by enhancing the penetration of drug molecules into the central nervous system and targeting key lesion sites. By leveraging the latest advances in nanotechnology, we may be able to overcome the significant challenges involved in treating CNS diseases and achieve significantly improved outcomes for patients [183]. In recent years, a diverse array of nanocarriers, including polymers, emulsions, lipid carriers, solid lipid carriers, carbon nanotubes, and metal-based carriers, have been utilized to develop highly effective therapies with sustained release properties. The incorporation of nanotechnology in drug delivery systems has significantly enhanced the bioavailability and pharmacokinetic properties of drugs in biological systems. For instance, improved bioenhancing effects have been demonstrated when curcumin is used in a nanocarrier [184]. With the aid of nanotechnology, drug delivery can be achieved in a safer and more targeted manner, enabling active molecules to reach specific sites of action and molecular targets with greater precision [185].

Nanoparticle technology serves as a robust foundation for the development of tissue-specific drug loading systems that are both highly versatile and customizable [186]. The implementation of nanodrug delivery systems with sustained release capabilities undoubtedly amplifies the controlled profile of the encapsulated drug. The range of nanoparticle varieties, such as polymeric NPs, solid lipid nanoparticles (SLNs), and liposomes, coupled with the ability to be paired with various macromolecules and exhibit an array of preferred physical and chemical properties, adds to their versatility. Neurological drugs prepared using nanomedical technology can maintain a high degree of bioavailability in the neural parenchyma after passing through the BBB. As a result, this advantage has stimulated significant curiosity and focus in the nanomedicine field [187]. At the same time, the characteristics of nanoparticles have inspired potential new strategies and minimally invasive approaches for the treatment and diagnosis of these neurological disorders.

As a cutting-edge therapeutic modality in novel drug delivery systems, nanomedicine harbours the capacity to surmount the diverse impediments associated with neurological disorders. Presently, over 20 nanomedicines have received clinical therapeutic or diagnostic approval from the FDA or the European Medicines Agency (EMA), and an additional 75 clinical trials examining nanoparticles have been initiated in recent years [188]. Although numerous nanomedicines approved for use or undergoing development are primarily designated for cancer treatments, these nanomedicines understandably reinforce the highly coveted and distinct features of nanomedicine and its immense biological potential for the management of neurological conditions [189]. Nanomedicine offers a viable solution to challenges that conventional medicines cannot address. Nanoparticles can be synthesized from a diverse range of materials and can be conjugated with other molecules, affording them exceptional design versatility. This characteristic translates to a host of favourable properties, including highly targeted tissue specificity, negligible immunogenicity, and superior stability in the bloodstream [190]. In the realm of neurological disorders, the most noteworthy benefit of nanocarrier systems is the independence of drug transport from the physical and chemical properties of NP-encapsulated drugs.

### Regulation of intestinal flora

Oral ingestion is the most convenient route for herbal medicine utilization, but first-pass metabolism in the liver and intestine adversely affects the effective conversion of active herbal ingredients. Recent years have witnessed the rapid advancement of advanced molecular technologies such as high-throughput sequencing and metabolomics. These techniques have revealed the close connection between the transformation of active ingredients of Chinese medicines and intestinal flora [191]. The intestinal flora plays a quintessential role in unlocking the full potential of Chinese traditional medicine and constitutes a critical step towards realizing the value of traditional medicine in China [192]. After oral administration, traditional Chinese medicine preparations come into contact with the gut microbiota and undergo mutual interactions, thereby achieving treatment efficacy through the regulation of the structure and metabolism of the gut microbiota [193]. Previous research has shown that herbal medicines exhibit the potential to regulate the metabolism of the gut microbiota by altering its composition, regulating its secretions and transforming herbal compounds [194]. The research team led by Liu Hongtao has combined microbiomics with Chinese medicine to investigate the pharmacological mechanisms of single and compound Chinese medicines in preventing and treating major diseases. Their work has shed light on the interactions between Chinese medicines and the host and intestinal flora. Notably, their findings have demonstrated that the crucial mechanism underlying the treatment of ulcerative colitis with oral 5-Amino Salicylic Acid (5-ASA) involves its regulatory effect on intestinal flora and bile acid metabolism [195].

The intestinal flora boasts formidable metabolic capacity and is rightfully regarded as a vital “metabolic organ” of the body. It performs crucial functions in various aspects of the host's well-being, such as digestion, nutrient absorption, metabolism, and immunity [196]. The potential biological significance of the gut flora in the development of depression has garnered considerable attention, given its vital role in gut-brain interactions. The intestinal flora not only impacts gut barrier function but also maintains host organism homeostasis by regulating the gut microbe-gut-brain (MGB) axis. The link between depression and gastrointestinal dysfunction is well documented, as appetite loss, metabolic issues, gastrointestinal dysfunction, and abnormal intestinal flora are observed in individuals with depression [197]. There is a clear relationship between the gut flora and depression, and MGB dysfunction may be one of the significant contributing factors to depression [198]. Chinese medicines possess a diverse range of medicinal properties that can regulate intestinal flora homeostasis within the body. They help to regulate the structure of the intestinal flora and to maintain the fundamental functions of the flora, thus alleviating the host’s disease state caused by flora disturbance or the secretion of harmful metabolites [192]. Poria cocos micropowder has been shown to alleviate depressive behaviour in rats by ameliorating the dysbiosis of intestinal flora caused by chronic unpredictable mild stress depression [199]. Additionally, Bupleuri Radix can effectively modulate the levels and composition of intestinal flora in chronic social defeat stress depression (CSDS) mice, promoting diversity and significantly improving their depression-like behaviour [200]. In a study conducted by Song Ruiwen et al., the impact of gallbladder-warming decoction on the abundance and composition of gut bacteria in rats subjected to a depression model was examined. The results indicated that gallbladder-warming decoction exhibited potential in regulating intestinal flora by enhancing the levels of probiotics and suppressing the growth of pathogenic bacteria, ultimately leading to an antidepressant effect [201]. In a study led by Ji Xuyan et al., the effects of Shunao Jieyu decoction on the gut microbiota of poststroke patients with depression were explored. The findings indicate that this herbal treatment successfully alleviated the clinical symptoms of poststroke depression according to Chinese medicine principles, reduced neurological impairment, enhanced daily living abilities, and modulated the diversity of gut microbiota in patients [202].

### Microenvironmental regulation methods

Microenvironmental modification is a rapidly developing research area that focuses on the environmental factors that impact drug action. This approach includes a variety of methods, ranging from site-specific alterations in the region of drug action to systemic administration of adjuvants that modify the host environment. As a comprehensive drug delivery strategy, microenvironmental regulation has the potential to improve drug bioavailability and effectiveness in diseased tissues by helping drugs penetrate biological barriers [203]. The blood‒brain barrier is a highly specialized structure that limits the movement of compounds between the blood and brain tissue. This barrier is created by a unique arrangement of components, including the nonporous endothelium of the brain capillaries, a continuous and unbroken basement membrane, and the perivascular astrocytic foot processes. These components possess varied permeability to different substances, which contributes to the selective transport of molecules into the brain [].

Modulating BBB permeability is a microenvironmental modification strategy aimed at selectively enhancing or restricting the passage of substances. The permeability of the blood‒brain barrier is primarily dictated by the integrity of the tight junctions between cerebral vascular endothelial cells, as well as the activity of efflux transporters such as P-glycoprotein (P-gp). Other factors, such as intercellular gaps, endocytosis, and transcytosis, also play a role in regulating BBB permeability [204]. Due to their fat solubility and aromatic properties, certain Kaigiao herbs, such as musk, borneol, and Acorus calamus, are able to readily cross the blood‒brain barrier. These herbs have been shown to inhibit the efflux of P-gp, resulting in increased drug concentrations within the brain. Additionally, these herbs have been found to suppress the expression of the protein Claudin-5, which widens the tight junctions between vascular endothelial cells, thereby enhancing BBB permeability. Acorus tatarinowii and borneol also increase the content of 5-hydroxy tryptamine (5-HT) and receptor binding rates, promoting the opening of tight junctions via endothelial cell contraction and increasing BBB permeability. Furthermore, Storesin and benzoin have been shown to increase BBB permeability by modulating P-gp exocytosis [168, 205]. Under pathological conditions, musk, borneol, Storesin, and benzoin have been found to reduce the content of inflammatory factors, which slows the damage of tight junctions between endothelial cells. Musk has also been shown to inhibit matrix metalloproteinase expression and mitigate cell damage caused by free radicals, promoting increased stability and reducing the permeability of the BBB and protecting brain tissues. Acorus calamus is capable of reducing BBB permeability through its anti-free radical properties, protecting brain tissues [206]. Cai Weiping’s study explored the effects of sodium hesperidin on the sarcoma homologue A (RhoA)/Rho-associated coiled-coil protein kinase (ROCK) pathway and BBB permeability in rats with bacterial meningitis (BM). The study demonstrated that sodium aescinate (SA) was able to improve brain oedema and BBB permeability in rats with BM. These beneficial effects of SA on BBB damage may be attributed to the inhibition of Rohan/ROCK pathway activation [170, 207].

## Concluding remarks and future perspective

In recent years, there has been a gradual increase in the incidence of depression. However, due to the unique structural characteristics of the BBB in neurological disorders, active drug molecules are unable to accumulate in brain lesions, severely impacting the effectiveness of treatment. Therefore, the search for novel and efficient drugs to treat brain diseases has become an urgent matter in today’s society. Domestic and international research has demonstrated that certain new drug delivery systems are capable of partially overcoming the BBB limitations and delivering drugs to focal areas in the brain, thus improving therapeutic effectiveness. The research and development of new systems have become a focus of clinical development. The aim of novel drug delivery systems, such as nanomedicines, liposomes, microneedles, and subcutaneous injections, is to improve drug efficacy and reduce side effects while enhancing patient compliance. These systems have advantages in terms of drug delivery, enhanced drug efficacy, controlled drug release, and other aspects and have achieved certain results in clinical development (Table 5).

**Table 5:** Novel drug delivery systems in clinical development

Treatment modality	System	Component	Example	References
Noninvasive	Controlled release capsule (medicine)	Small molecule compounds	Hawthorn Leaf Total Flavonoids Extended-Release Capsules	[208]
	Inhalable devices	Compound prescription (Chinese medicine)	Nebulized inhalation of Chinese medicine	[209]
	Liposome	Small molecule compounds	Honokiol Liposome lyophilized powder	[210]
	Nanoparticles	Small molecule compounds	Astragalus polysaccharide nanotumor in situ vaccine	[211]
	Transdermal patches	Monomers of Chinese medicine	Chinese Medicine Acupuncture Point Patching	[212]
Invasive	Microneedle	Compound prescription (Chinese medicine)	“Jade Face” soluble microneedle	[213]
	Implant	Monomers of Chinese medicine	Epimedium extract nanotube biomimetic coating	[214]
	Microsphere	Small molecule compounds	Huperzine A Slow-release microspheres	[215]
	Liposome/nanoparticles	Small molecule compounds	Ursolic acid nanoliposomes; Paclitaxel liposome	[216, 217]
	Hypodermic injection	Small molecule compounds	Paclitaxel polymer micelles for injection	[218]

NDDSs are the foundation of drug formulation research and play a pivotal role in the development of the global pharmaceutical industry. The focus of NDDS research is to optimize drug efficacy and reduce unwanted side effects by implementing three fundamental strategies: targeting space, release time, and dose. The scope of NDDSs encompasses various objectives, such as controlled drug release, drug targeting, enhanced drug stability, the regulation of drug metabolism time, improved drug absorption, and overcoming biological barriers.

The cornerstone of modernizing Chinese medicine lies in the modernization of Chinese medicine formulations. By harnessing the advantages of traditional Chinese medicine in treating brain diseases and integrating modern new drug delivery systems, Chinese medicine can effectively treat brain diseases and the dosage forms of Chinese medicines can be improved. The key factors affecting the ability of a drug to cross the BBB include its lipid solubility, molecular weight, and dissociated or nondissociated form under physiological conditions. NDDSs have demonstrated the ability to enhance the ability of Chinese medicines to penetrate the BBB and increase drug concentrations in the brain. This combination of strengths minimizes the adverse effects of Chinese medicines while maximizing their therapeutic benefits.

In conclusion, the vast potential of Chinese medicine remains largely untapped. Outdated drug delivery methods have limited the efficacy of Chinese medicines. The application of new drug delivery technologies to Chinese herbal medicines will undoubtedly help to enhance the therapeutic efficacy of various Chinese medicinal compounds and herbs while reducing the side effects of drugs. The progress made by modern science has provided additional opportunities to explore the benefits of Chinese medicine. While new drug delivery systems have shown promise in treating brain diseases, they are still in their early stages, and numerous challenges are encountered. Further research is necessary to refine drug delivery carriers and optimize TCM formulations.

## Data Availability

The raw data supporting the conclusions of this manuscript will be made available by the authors, without undue reservation, to any qualified researcher.

## Abbreviations

ADA: Adenosine deaminase
ADV: Adenoviruses
ADA-SCID: Adenosine deaminase Severe combined immune deficiency
API: Active pharmaceutical ingredient
5-ASA: 5-Amino Salicylic Acid
BBB: Blood brain barrier
BCECs: Brain capillary endothelial cells
BM: Bacterial meningitis
Chronic CSDS: Social defeat stress depression
DDS: Drug delivery systems
D2: Dopamine2
EMA: European Medicines Agency
EGFR: Epidermal growth factor receptor
Gen: Genistein
LV: Lentiviruses
MGB: Microbe-gut-brain
MAO: Monoamine oxidase
mAbs: Monoclonal antibodies
NSCLC: Non-small cell lung cancer
NDDS: Novel drug delivery systems
PK: Pharmacokinetic
PEG-PLGA: Polyethylene glycol-poly lactic acid-hydroxyacetic acid
PLGA: Poly lactic-co-glycolic acid
P-gp: P-glycoprotein
ROCK: Rho-associated coiled-coil protein kinase
RV: Retroviruses
SLNs: Solid lipid nanoparticles
SA: Sodium aescinate
SARS-CoV-2: Severe Acute Respiratory Syndrome Coronavirus 2
TCM: Traditional Chinese Medicine
TEER: Transendothelial resistance

## References

[1] Smith K (2014). Mental health: a world of depression. Nature.

[2] Feng ST, Wang XL, Wang YT, Yuan YH, Li ZP, Chen NH, Wang ZZ, Zhang Y (2021). Efficacy of Traditional chinese medicine combined with selective serotonin reuptake inhibitors on the treatment for Parkinson’s disease with depression: a systematic review and meta-analysis. Am J Chin Med.

[3] Zhang Y, Wang ZZ, Sun HM, Li P, Li YF, Chen NH (2014). Systematic review of traditional chinese medicine for depression in Parkinson's disease. Am J Chin Med.

[4] Zhang G, Xiong N, Zhang Z, Liu L, Huang J, Yang J, Wu J, Lin Z, Wang T (2015). Effectiveness of traditional Chinese medicine as an adjunct therapy for Parkinson's disease: a systematic review and meta-analysis. PLoS ONE.

[5] Cheng G, Liu Y, Ma R, Cheng G, Guan Y, Chen X, Wu Z, Chen T (2022). Anti-Parkinsonian therapy: strategies for crossing the blood-brain barrier and nano-biological effects of nanomaterials. Nanomicro Lett.

[6] Gao H (2016). Progress and perspectives on targeting nanoparticles for brain drug delivery. Acta Pharm Sin B.

[7] Daneman R, Prat A (2015). The blood-brain barrier. Cold Spring Harb Perspect Biol.

[8] Reeve A, Simcox E, Turnbull D (2014). Ageing and Parkinson's disease: why is advancing age the biggest risk factor?. Ageing Res Rev.

[9] Neuwelt E, Abbott NJ, Abrey L, Banks WA, Blakley B, Davis T, Engelhardt B, Grammas P, Nedergaard M, Nutt J (2008). Strategies to advance translational research into brain barriers. Lancet Neurol.

[10] Nagpal K, Singh SK, Mishra DN (2013). Drug targeting to brain: a systematic approach to study the factors, parameters and approaches for prediction of permeability of drugs across BBB. Expert Opin Drug Deliv.

[11] Pardridge WM (2003). Blood-brain barrier drug targeting: the future of brain drug development. Mol Interv.

[12] Chen Y, Liu L (2012). Modern methods for delivery of drugs across the blood-brain barrier. Adv Drug Deliv Rev.

[13] Takata F, Nakagawa S, Matsumoto J, Dohgu S (2021). Blood-brain barrier dysfunction amplifies the development of neuroinflammation: understanding of cellular events in brain microvascular endothelial cells for prevention and treatment of BBB dysfunction. Front Cell Neurosci.

[14] Zlokovic BV (2008). The blood-brain barrier in health and chronic neurodegenerative disorders. Neuron.

[15] Daneman R (2012). The blood-brain barrier in health and disease. Ann Neurol.

[16] Profaci CP, Munji RN, Pulido RS, Daneman R (2020). The blood-brain barrier in health and disease: important unanswered questions. J Exp Med.

[17] Tosi G, Duskey JT, Kreuter J (2020). Nanoparticles as carriers for drug delivery of macromolecules across the blood-brain barrier. Expert Opin Drug Deliv.

[18] Wang J, Tang W, Yang M, Yin Y, Li H, Hu F, Tang L, Ma X, Zhang Y, Wang Y (2021). Inflammatory tumor microenvironment responsive neutrophil exosomes-based drug delivery system for targeted glioma therapy. Biomaterials.

[19] Mingzhao Z (2001). Beyond the traditional route of administration. Foreign Sci Technol Trends.

[20] Mc Gillicuddy A, Crean AM, Sahm LJ (2016). Older adults with difficulty swallowing oral medicines: a systematic review of the literature. Eur J Clin Pharmacol.

[21] Kaur G, Arora M, Ravi Kumar MNV (2019). Oral drug delivery technologies: a decade of developments. J Pharmacol Exp Ther.

[22] McConville JT (2017). Current developments in oral drug administration. Drug Dev Ind Pharm.

[23] Öztürk AA, Arpagaus C (2021). Nano Spray-Dried Drugs for Oral Administration: A Review.. Chin J Tradit Chin Med..

[24] Vargason AM, Anselmo AC, Mitragotri S (2021). The evolution of commercial drug delivery technologies. Nat Biomed Eng.

[25] Bae YH, Park K (2020). Advanced drug delivery 2020 and beyond: Perspectives on the future. Adv Drug Deliv Rev.

[26] Martinho N, Damgé C, Reis CP (2011). Recent advances in drug delivery systems. J Biomater Nanobiotechnology.

[27] Jain KK (2008). Drug delivery systems.

[28] Zhuang W, Liu S-L, Xi S-Y, Feng Y-N, Wang K, Abduwali T, Liu P, Zhou X-J, Zhang L, Dong X-Z (2023). Traditional Chinese medicine decoctions and Chinese patent medicines for the treatment of depression: Efficacies and mechanisms. J Ethnopharmacol.

[29] Li Ruyue DG, Xinyi Y (2022). Study on pharmacodynamics and pharmacokinetics of Hypericum perforatum combined with sertraline in depression model rats. Jiangsu Trad Chin Med.

[30] Van Hoogdalem EJ, de Boer AG, Breimer DD. Pharmacokinetics of rectal drug administration, Part II. Clinical applications of peripherally acting drugs, and conclusions. Clin Pharmacokinet. 1991; 21:110–128. 10.2165/00003088-199121020-00003 1884566

[31] Shen Shi W, Song H (2023). Regulatory effect and mechanism of magnesium baicalin on depression-like behavior and neuroinflammation induced by lipopolysaccharide in rats. China J Pharm..

[32] Zhao Zhiyu WW, Guo H (2006). Effect of liquiritin on weight and behavior of depression model rats. Chin Mental Health J..

[33] Zou Yuchi CJ, Huang W (2019). Effects of curcumin analogue L6H3 on monoamine neurotransmitter metabolites in striatum of Parkinson's disease rats. J Wenzhou Med Univ..

[34] Zhang T-T, Jiang J-G (2012). Active ingredients of traditional Chinese medicine in the treatment of diabetes and diabetic complications. Expert Opin Investig Drugs.

[35] Liu X, Shi D, Zhou S, Liu H, Liu H, Yao X (2018). Molecular dynamics simulations and novel drug discovery. Expert Opin Drug Discov.

[36] Huwyler J, Wu D, Pardridge WM (1996). Brain drug delivery of small molecules using immunoliposomes. Proc Natl Acad Sci.

[37] Wang A-A, Li L, Li Y, Miao L, Pan Y-H, Liu J-X (2021). Research progress on Chinese medicinal material-derived active polypeptides against ischemic cardiovascular and cerebrovascular diseases. Zhongguo Zhong Yao Za Zhi.

[38] Shi Zhongkai HX, You Y (2011). Experimental** study on anti-tumor activity of ethanol extract from roots of Linqian on S-180 tumor-bearing mice**. Journal of Chengdu University of Traditional Chinese Medicine..

[39] Baolan S (2014). Research progress of anti-liver injury traditional Chinese medicine and its mechanism of action. Everybody's Health (Academic Edition).

[40] Shen Shi W GX, Song Hongru. Regulatory effect and mechanism of magnesium baicalin on depression-like behavior and neuroinflammation induced by lipopolysaccharide in rats. China Journal of Pharmacy. 2023;58:338–346.

[41] Alam MI, Beg S, Samad A, Baboota S, Kohli K, Ali J, Ahuja A, Akbar M (2010). Strategy for effective brain drug delivery. Eur J Pharm Sci.

[42] Ma G (2014). Microencapsulation of protein drugs for drug delivery: strategy, preparation, and applications. J Control Release.

[43] Li C, Wang J, Wang Y, Gao H, Wei G, Huang Y, Yu H, Gan Y, Wang Y, Mei L (2019). Recent progress in drug delivery. Acta Pharm Sin B.

[44] Liechty WB, Kryscio DR, Slaughter BV, Peppas NA (2010). Polymers for drug delivery systems. Annu Rev Chem Biomol Eng.

[45] Manzari MT, Shamay Y, Kiguchi H, Rosen N, Scaltriti M, Heller DA (2021). Targeted drug delivery strategies for precision medicines. Nat Rev Mater.

[46] Cai SS, Li T, Akinade T, Zhu Y, Leong KW (2021). Drug delivery carriers with therapeutic functions. Adv Drug Deliv Rev.

[47] He Ying FF, Yan Hongli. Momordica charantia MAP30 promotes autophagy and apoptosis of multiple myeloma cells through AKT/mTOR pathway. chinese journal of cancer biotherapy. 2019; 26:299–305.

[48] Tewabe A, Abate A, Tamrie M, Seyfu A, Abdela Siraj E (2021). Targeted drug delivery—from magic bullet to nanomedicine: principles, challenges, and future perspectives. J Multidiscip Healthc.

[49] Hakim LK, Yazdanian M, Alam M, Abbasi K, Tebyaniyan H, Tahmasebi E, Khayatan D, Seifalian A, Ranjbar R, Yazdanian A (2021). Biocompatible and biomaterials application in drug delivery system in oral cavity. Evid Based Complement Alternat Med.

[50] Alqahtani MS, Kazi M, Alsenaidy MA, Ahmad MZ (2021). Advances in oral drug delivery. Front Pharmacol.

[51] Haywood A, Glass BD (2011). Pharmaceutical excipients–where do we begin?. Aust Prescr.

[52] Am Ende MT (2019). Chemical engineering in the pharmaceutical industry: active pharmaceutical ingredients.

[53] Zompra AA, Galanis AS, Werbitzky O, Albericio F (2009). Manufacturing peptides as active pharmaceutical ingredients. Future Med Chem.

[54] Park H, Otte A, Park K (2022). Evolution of drug delivery systems: From 1950 to 2020 and beyond. J Control Release.

[55] Barkat MA, Rizwanullah M, Pottoo FH, Beg S, Akhter S, Ahmad FJ (2020). Therapeutic nanoemulsion: concept to delivery. Curr Pharm Des.

[56] Lu XY, Wu DC, Li ZJ, Chen GQ (2011). Polymer nanoparticles. Prog Mol Biol Transl Sci.

[57] Elvira C, Gallardo A, Roman JS, Cifuentes A (2005). Covalent polymer-drug conjugates. Molecules.

[58] Dang W, Colvin OM, Brem H, Saltzman WM (1994). Covalent coupling of methotrexate to dextran enhances the penetration of cytotoxicity into a tissue-like matrix. Cancer Res.

[59] Guan Qingxia HX, Li W (2015). Research progress of liver-targeted nano-drug delivery system loaded with traditional Chinese medicine. China Pharm.

[60] Alqahtani MS, Kazi M, Alsenaidy MA, Ahmad MZ. Advances in oral drug delivery. Frontiers in pharmacology.2021;12:618411–618417. 10.3389/fphar.2021.618411 PMC7933596 33679401

[61] Duan Xiaoying JQ, Liu L (2017). Preparation of Matrine Nanoparticles and Modified Products of Wheat Germ Lectin. Chin Patent Med.

[62] Harwansh RK, Deshmukh R, Rahman MA (2019). Nanoemulsion: Promising nanocarrier system for delivery of herbal bioactives. J Drug Deliv Sci Technol.

[63] Zhang Yanfang GY, Wu R (2020). Research progress of Chinese medicine extract nanoemulsion and its pharmacokinetics. China J Chin Med Inform.

[64] Kotta S, Aldawsari HM, Badr-Eldin SM, Alhakamy NA, Md S (2021). Coconut oil-based resveratrol nanoemulsion: optimization using response surface methodology, stability assessment and pharmacokinetic evaluation. Food Chem.

[65] Mohamed S, Parayath NN, Taurin S, Greish K (2014). Polymeric nano-micelles: versatile platform for targeted delivery in cancer. Ther Deliv.

[66] Hwang D, Ramsey JD, Kabanov AV (2020). Polymeric micelles for the delivery of poorly soluble drugs: from nanoformulation to clinical approval. Adv Drug Deliv Rev.

[67] Gill KK, Kaddoumi A, Nazzal S (2012). Mixed micelles of PEG(2000)-DSPE and vitamin-E TPGS for concurrent delivery of paclitaxel and parthenolide: enhanced chemosenstization and antitumor efficacy against non-small cell lung cancer (NSCLC) cell lines. Eur J Pharm Sci.

[68] Shah S, Dhawan V, Holm R, Nagarsenker MS, Perrie Y (2020). Liposomes: advancements and innovation in the manufacturing process. Adv Drug Deliv Rev.

[69] Almeida B, Nag OK, Rogers KE, Delehanty JB (2020). Recent progress in bioconjugation strategies for liposome-mediated drug delivery. Molecules.

[70] Guimarães D, Cavaco-Paulo A, Nogueira E (2021). Design of liposomes as drug delivery system for therapeutic applications. Int J Pharm.

[71] Yan Dekang ZX, Wang Y (2022). Formulation optimization of mannose modified curcumin/ginsenoside Rb1 liposomes and evaluation of brain targeting in vitro. China Pharm.

[72] Walther R, Rautio J, Zelikin AN (2017). Prodrugs in medicinal chemistry and enzyme prodrug therapies. Adv Drug Deliv Rev.

[73] Kang T, Miao Z, Liu S, Ke B (2021). Prodrug strategies in the CNS drugs: small modification makes big improvements. Curr Top Med Chem.

[74] Chen KJ, Plaunt AJ, Leifer FG, Kang JY, Cipolla D (2021). Recent advances in prodrug-based nanoparticle therapeutics. Eur J Pharm Biopharm.

[75] Abet V, Filace F, Recio J, Alvarez-Builla J, Burgos C (2017). Prodrug approach: an overview of recent cases. Eur J Med Chem.

[76] Mehellou Y, Rattan HS, Balzarini J (2018). The ProTide prodrug technology: from the concept to the clinic. J Med Chem.

[77] Jana S, Mandlekar S, Marathe P (2010). Prodrug design to improve pharmacokinetic and drug delivery properties: challenges to the discovery scientists. Curr Med Chem.

[78] Bodor N, Kaminski JJ (1987). Prodrugs and site-specific chemical delivery systems. Ann Rep Med Chem.

[79] Chen Juan PL, Yang Xiaoyi. Analysis of global research and development situation of virus vector vaccine products. China Pharmaceutical. 2022; 31:24–29.

[80] He F, Wen N, Xiao D, Yan J, Xiong H, Cai S, Liu Z, Liu Y (2020). Aptamer-based targeted drug delivery systems: current potential and challenges. Curr Med Chem.

[81] Liu P, Jiang C (2022). Brain-targeting drug delivery systems. Wiley Interdiscip Rev Nanomed Nanobiotechnol.

[82] Shah V, Kochar P (2018). Brain cancer: implication to disease, therapeutic strategies and tumor targeted drug delivery approaches. Recent Pat Anticancer Drug Discov.

[83] Morachis JM, Mahmoud EA, Almutairi A (2012). Physical and chemical strategies for therapeutic delivery by using polymeric nanoparticles. Pharmacol Rev.

[84] Tian B, Hua S, Tian Y, Liu J (2020). Chemical and physical chitosan hydrogels as prospective carriers for drug delivery: a review. J Mater Chem B.

[85] Haiyan H (2015). Research progress of magnetic targeting in physicochemical targeting preparations. Guangzhou Chem Ind.

[86] Polyak B, Friedman G (2009). Magnetic targeting for site-specific drug delivery: applications and clinical potential. Expert Opin Drug Deliv.

[87] Bietenbeck M, Florian A, Faber C, Sechtem U, Yilmaz A (2016). Remote magnetic targeting of iron oxide nanoparticles for cardiovascular diagnosis and therapeutic drug delivery: where are we now?. Int J Nanomedicine.

[88] Yan Runmin LC, Zhao M (2011). Experimental study on distribution of magnetic paclitaxel-ferroferric oxide-drug-loaded liposome complex particles in brain. Biomed Eng Clin Med.

[89] Peng N, Yu H, Yu W, Yang M, Chen H, Zou T, Deng K, Huang S, Liu Y (2020). Sequential-targeting nanocarriers with pH-controlled charge reversal for enhanced mitochondria-located photodynamic-immunotherapy of cancer. Acta Biomater.

[90] Chen Conghui LM (2019). Anti-tumor study of targeting and pH-sensitive lipoprotein-like nano-carriers carrying paclitaxel in vivo. China J Trad Chin Med.

[91] Shirley JL, de Jong YP, Terhorst C, Herzog RW (2020). Immune responses to viral gene therapy vectors. Mol Ther.

[92] Anderson WF (1984). Prospects for human gene therapy. Science.

[93] Milone MC, O'Doherty U (2018). Clinical use of lentiviral vectors. Leukemia.

[94] Jolly D (1994). Viral vector systems for gene therapy. Cancer Gene Ther.

[95] Thomas CE, Ehrhardt A, Kay MA (2003). Progress and problems with the use of viral vectors for gene therapy. Nat Rev Genet.

[96] Halperin SA, Ye L, MacKinnon-Cameron D, Smith B, Cahn PE, Ruiz-Palacios GM, Ikram A, Lanas F, Lourdes Guerrero M, Muñoz Navarro SR (2022). Final efficacy analysis, interim safety analysis, and immunogenicity of a single dose of recombinant novel coronavirus vaccine (adenovirus type 5 vector) in adults 18 years and older: an international, multicentre, randomised, double-blinded, placebo-controlled phase 3 trial. Lancet.

[97] Zhang YH, Wang Y, Yusufali AH, Ashby F, Zhang D, Yin ZF, Aslanidi GV, Srivastava A, Ling CQ, Ling C (2014). Cytotoxic genes from traditional Chinese medicine inhibit tumor growth both in vitro and in vivo. J Integr Med.

[98] Raposo G, Stahl PD (2019). Extracellular vesicles: a new communication paradigm?. Nat Rev Mol Cell Biol.

[99] Söllner T, Whiteheart SW, Brunner M, Erdjument-Bromage H, Geromanos S, Tempst P, Rothman JE (1993). SNAP receptors implicated in vesicle targeting and fusion. Nature.

[100] Elsharkasy OM, Nordin JZ, Hagey DW, de Jong OG, Schiffelers RM, Andaloussi SE, Vader P (2020). Extracellular vesicles as drug delivery systems: why and how?. Adv Drug Deliv Rev.

[101] Zhao L, Hu C, Han F, Wang J, Chen J (2020). Regenerative abilities of mesenchymal stem cells via acting as an ideal vehicle for subcellular component delivery in acute kidney injury. J Cell Mol Med.

[102] de Castilla PEM, Tong L, Huang C, Sofias AM, Pastorin G, Chen X, Storm G, Schiffelers RM, Wang J-W (2021). Extracellular vesicles as a drug delivery system: a systematic review of preclinical studies. Adv Drug Deliv Rev.

[103] Zhu Q, Ling X, Yang Y, Zhang J, Li Q, Niu X, Hu G, Chen B, Li H, Wang Y, Deng Z (2019). Embryonic stem cells-derived exosomes endowed with targeting properties as chemotherapeutics delivery vehicles for glioblastoma therapy. Adv Sci (Weinh).

[104] Villa F, Quarto R, Tasso R (2019). Extracellular vesicles as natural, safe and efficient drug delivery systems. Pharmaceutics.

[105] Zou J, Shi M, Liu X, Jin C, Xing X, Qiu L, Tan W (2019). Aptamer-functionalized exosomes: elucidating the cellular uptake mechanism and the potential for cancer-targeted chemotherapy. Anal Chem.

[106] Xi X-M, Xia S-J, Lu R (2021). Drug loading techniques for exosome-based drug delivery systems. Die Pharm.

[107] Ajazuddin S (2010). Applications of novel drug delivery system for herbal formulations. Fitoterapia.

[108] Thwala LN, Préat V, Csaba NS (2017). Emerging delivery platforms for mucosal administration of biopharmaceuticals: a critical update on nasal, pulmonary and oral routes. Expert Opin Drug Deliv.

[109] Naik A, Kalia YN, Guy RH (2000). Transdermal drug delivery: overcoming the skin's barrier function. Pharm Sci Technol Today.

[110] Mavuso S, Marimuthu T, Choonara YE, Kumar P, du Toit LC, Pillay V (2015). A review of polymeric colloidal nanogels in transdermal drug delivery. Curr Pharm Des.

[111] Lan Y, Wang J, Li H, Zhang Y, Chen Y, Zhao B, Wu Q (2016). Effect of menthone and related compounds on skin permeation of drugs with different lipophilicity and molecular organization of stratum corneum lipids. Pharm Dev Technol.

[112] Balfour DJ, Fagerström KO (1996). Pharmacology of nicotine and its therapeutic use in smoking cessation and neurodegenerative disorders. Pharmacol Ther.

[113] Rezvani AH, Levin ED (2001). Cognitive effects of nicotine. Biol Psychiatry.

[114] Singh N, Pillay V, Choonara YE (2007). Advances in the treatment of Parkinson's disease. Prog Neurobiol.

[115] Shen Chengying SB, Xu P (2014). Preparation of Ganoderma lucidum triterpenoid nanosuspension gel and its transdermal study in vitro. Chinese Herb Med.

[116] Siegel SJ, Winey KI, Gur RE, Lenox RH, Bilker WB, Ikeda D, Gandhi N, Zhang WX (2002). Surgically implantable long-term antipsychotic delivery systems for the treatment of schizophrenia. Neuropsychopharmacology.

[117] Rabin C, Liang Y, Ehrlichman RS, Budhian A, Metzger KL, Majewski-Tiedeken C, Winey KI, Siegel SJ (2008). In vitro and in vivo demonstration of risperidone implants in mice. Schizophr Res.

[118] Wang Z, Xiong G, Tsang WC, Schätzlein AG, Uchegbu IF (2019). Nose-to-brain delivery. J Pharmacol Exp Ther.

[119] Lobaina Mato Y (2019). Nasal route for vaccine and drug delivery: features and current opportunities. Int J Pharm.

[120] Kashyap K, Shukla R (2019). Drug delivery and targeting to the brain through nasal route: mechanisms, applications and challenges. Curr Drug Deliv.

[121] Dongyuan L (2016). Theory of qi alternation in five organs. Bull Trad Chin Med.

[122] Hou H, Li Y, Xu Z, Yu Z, Peng B, Wang C, Liu W, Li W, Ye Z, Zhang G (2023). Applications and research progress of traditional Chinese medicine delivered via nasal administration. Biomed Pharmacother.

[123] Yang CX, Xu XH, Dong Y (2003). Advances in the research on targeted preparations of traditional Chinese medicine and natural drugs. Zhongguo Zhong Yao Za Zhi.

[124] Ju Aichun GS, Yang X (2017). Effects of nasal administration of salvianolic acid B on learning and memory ability and nerve regeneration in rats with cerebral ischemia injury. Chin Herbal Med.

[125] Sun Yaping ZY, Bai N (2018). Effect of short-term nasal administration of genistein on alleviating neuronal damage after cerebral infarction in rats. Chin J Coal Ind Med.

[126] Guo Q, Li P, Wang Z, Cheng Y, Wu H, Yang B, Du S, Lu Y (2014). Brain distribution pharmacokinetics and integrated pharmacokinetics of* Panax** Notoginsenoside* R1, ginsenosides Rg1, Rb1, Re and Rd in rats after intranasal administration of* Panax** Notoginseng* Saponins assessed by UPLC/MS/MS. J Chromatogr B Analyt Technol Biomed Life Sci.

[127] Dong Y, Shi J-R (2012). Biological research evaluating the Chinese medical theory of the association of the kidney with the ears. Zhong Xi Yi Jie He Xue Bao.

[128] Zhu Q, Ling X, Yang Y, Zhang J, Li Q, Niu X, Hu G, Chen B, Li H, Wang Y, Deng Z. Embryonic Stem Cells-Derived Exosomes Endowed with Targeting Properties as Chemotherapeutics Delivery Vehicles for Glioblastoma Therapy. Adv Sci (Weinh). 2019; 6:1809–1899. 10.1002/advs.201801899 PMC6425428 30937268

[129] Villa F, Quarto R, Tasso R. Extracellular vesicles as natural, safe and efficient drug delivery systems. Pharmaceutics. 2019; 11:557–561. 10.3390/pharmaceutics11110557 PMC6920944 31661862

[130] An Xiaogang CD (2020). Research progress of nano-particle drug delivery system in inner ear targeted drug delivery. Chin J Otol.

[131] Jiang W-Y (2005). Therapeutic wisdom in traditional Chinese medicine: a perspective from modern science. Trends Pharmacol Sci.

[132] Liu H, Hao J, Li KS (2013). Current strategies for drug delivery to the inner ear. Acta Pharm Sin B.

[133] Qiu K, Mao M, Deng D, Jiang C, Li L, Zheng Y, Ren J, Zhao Y (2022). Is postauricular injection a systemic or a topical route for inner ear drug delivery?. Hear Res.

[134] Lee S, Dondzillo A, Gubbels SP, Raphael Y (2020). Practical aspects of inner ear gene delivery for research and clinical applications. Hear Res.

[135] Xue Yaqi WZ, Liang Peiyi. Progress in molecular pharmaceutics of transdermal preparations of traditional Chinese medicine. Journal of Nanjing University of Traditional Chinese Medicine. 2022; 38:983–989.

[136] Pferschy-Wenzig E-M, Bauer R (2015). The relevance of pharmacognosy in pharmacological research on herbal medicinal products. Epilepsy Behav.

[137] Jingjing W, Rongjuan G, Guojing F, Xiao Liang ZX, Min J, Zixiu Z, Wanqing D, Weiwei J, Linjuan S, Hongmei L (2022). Registration of intervention trials of traditional Chinese medicine for four neurological diseases on Chinese clinical trial registry and ClinicalTrials.gov: a narrative review. J Trad Chin Med.

[138] Liu Y, Feng N (2015). Nanocarriers for the delivery of active ingredients and fractions extracted from natural products used in traditional Chinese medicine (TCM). Adv Coll Interface Sci.

[139] Verma H, Prasad SB, Yashwant SH (2013). Herbal drug delivery system: a modern era prospective. Int J Current Pharma Rev Res.

[140] Dement'eva OV, Naumova KA, Zhigletsova SK, Klykova MV, Somov AN, Dunaytsev IA, Senchikhin IN, Volkov VV, Rudoy VM (2020). Drug-templated mesoporous silica nanocontainers with extra high payload and controlled release rate. Colloids Surf B Biointerfaces.

[141] Davoodi P, Lee LY, Xu Q, Sunil V, Sun Y, Soh S, Wang CH (2018). Drug delivery systems for programmed and on-demand release. Adv Drug Deliv Rev.

[142] Yang D, Lee JS, Choi CK, Lee HP, Cho SW, Ryu W (2018). Microchannel system for rate-controlled, sequential, and pH-responsive drug delivery. Acta Biomater.

[143] Severino P, da Silva CF, Andrade LN, de Lima OD, Campos J, Souto EB (2019). Alginate nanoparticles for drug delivery and targeting. Curr Pharm Des.

[144] Torchilin VP (2000). Drug targeting. Eur J Pharm Sci.

[145] Jain K (2007). Nanobiotechnology-based drug delivery to the central nervous system. Neurodegener Dis.

[146] Ipar VS, Dsouza A, Devarajan PV (2019). Enhancing curcumin oral bioavailability through nanoformulations. Eur J Drug Metab Pharmacokinet.

[147] Blasi P, Giovagnoli S, Schoubben A, Ricci M, Rossi C (2007). Solid lipid nanoparticles for targeted brain drug delivery. Adv Drug Deliv Rev.

[148] Kumari A, Yadav SK, Yadav SC (2010). Biodegradable polymeric nanoparticles based drug delivery systems. Colloids Surf B Biointerfaces.

[149] Silva GA (2008). Nanotechnology approaches to crossing the blood-brain barrier and drug delivery to the CNS. BMC Neurosci.

[150] Anselmo AC, Mitragotri S (2019). Nanoparticles in the clinic: an update. Bioeng Transl Med.

[151] Havel H, Finch G, Strode P, Wolfgang M, Zale S, Bobe I, Youssoufian H, Peterson M, Liu M (2016). Nanomedicines: from bench to bedside and beyond. AAPS J.

[152] Silva GA (2010). Nanotechnology applications and approaches for neuroregeneration and drug delivery to the central nervous system. Ann NY Acad Sci.

[153] Huo Liang PY, Liu X (2022). Research progress of intestinal flora on metabolism of effective components of traditional Chinese medicine. Chin J Trad Chin Med.

[154] Zhang Qi JY, Zheng HC, Zhao CB (2022). Research progress on the interaction between metabolism of effective components of traditional Chinese medicine and intestinal flora. Northw Pharm J.

[155] Yuting J (2021). Research progress of traditional Chinese medicine in regulating intestinal flora changes of colorectal cancer. Clin Res Trad Chin Med.

[156] Ke Qunhua PJ, Wang S (2022). Research progress on traditional Chinese medicine and intestinal flora and its metabolism. J Trad Chin Vet Med..

[157] Huang L, Zheng J, Sun G, Yang H, Sun X, Yao X, Lin A, Liu H (2022). 5-Aminosalicylic acid ameliorates dextran sulfate sodium-induced colitis in mice by modulating gut microbiota and bile acid metabolism. Cell Mol Life Sci.

[158] Bi Huichang YM (2020). Intestinal flora and drug metabolism. Pharm Prog.

[159] Jiang H, Ling Z, Zhang Y, Mao H, Ma Z, Yin Y, Wang W, Tang W, Tan Z, Shi J (2015). Altered fecal microbiota composition in patients with major depressive disorder. Brain Behav Immun.

[160] Dong Y, Shi J-R. Biological research evaluating the Chinese medical theory of the association of the kidney with the ears. Zhong xi yi jie he xue bao= Journal of Chinese Integrative Medicine. 2012; 10:128–134. 10.3736/jcim20120202 22313879

[161] Xu Yan LD, Ning T (2021). Effects of Poria cocos on behavior and fecal flora in rats with mild and chronic unpredictable stress. China Pharmacovigil.

[162] Cai Saibo ZH, Xin X (2021). Effect of taking Bupleurum chinense DC. on intestinal flora diversity in depressed mice. China J Trad Chin Med..

[163] Song Ruiwen ZL, Wang S (2021). Analysis of the effect of Jiawei Wendan Decoction on intestinal flora of depression model rats based on 16S rDNA high-throughput sequencing technique. J Trad Chin Med..

[164] Ji Xuyan HZ, Li T (2022). Effect of Shunao Jieyu decoction on intestinal flora in patients with post-stroke depression. Chin J Exp Trad Med Formulae..

[165] Hou S, Gao Y-E, Ma X, Lu Y, Li X, Cheng J, Wu Y, Xue P, Kang Y, Guo M (2021). Tumor microenvironment responsive biomimetic copper peroxide nanoreactors for drug delivery and enhanced chemodynamic therapy. Chem Eng J.

[166] Shao K, Zhang Y, Ding N, Huang S, Wu J, Li J, Yang C, Leng Q, Ye L, Lou J (2015). Functionalized nanoscale micelles with brain targeting ability and intercellular microenvironment biosensitivity for anti-intracranial infection applications. Adv Healthcare Mater.

[167] David A (2017). Peptide ligand-modified nanomedicines for targeting cells at the tumor microenvironment. Adv Drug Deliv Rev.

[168] gang C. construction of compound inner ear drug delivery system of traditional Chinese medicine and study on drug delivery mechanism. 2017;48:32–36

[169] Liu Chao LJ, Liu S (2016). Study on the mechanism of regulating blood-brain barrier by aromatic resuscitation drugs and the treatment of encephalopathy. J Changchun Univ Trad Chin Med..

[170] Cai Weiping CY, Tang L (2022). Effects of sodium aescinate on RhoA/ROCK pathway and blood-brain barrier permeability in rats with bacterial meningitis. China J Microecol.

[171] Öztürk AA, Arpagaus C (2021). Nano spray-dried drugs for oral administration: a review. Assay Drug Dev Technol.

[172] Nielsen LH, Keller SS, Boisen A (2018). Microfabricated devices for oral drug delivery. Lab Chip.

[173] Pennington CA, Park JM (2015). Sublingual tacrolimus as an alternative to oral administration for solid organ transplant recipients. Am J Health Syst Pharm.

[174] Goswami T, Jasti B, Li X (2008). Sublingual drug delivery. Crit Rev Ther Drug Carrier Syst.

[175] Usach I, Martinez R, Festini T, Peris JE (2019). Subcutaneous injection of drugs: literature review of factors influencing pain sensation at the injection site. Adv Ther.

[176] Logomasini MA, Stout RR, Marcinkoski R (2013). Jet injection devices for the needle-free administration of compounds, vaccines, and other agents. Int J Pharm Compd.

[177] Frid AH, Hirsch LJ, Menchior AR, Morel DR, Strauss KW (2016). Worldwide injection technique questionnaire study: injecting complications and the role of the professional. Mayo Clin Proc.

[178] van Hoogdalem EJ, de Boer AG, Breimer DD (1991). Pharmacokinetics of rectal drug administration, Part II. Clinical applications of peripherally acting drugs, and conclusions. Clin Pharmacokinet.

[179] Lowry M (2016). Rectal drug administration in adults: how, when, why. Nurs Times.

[180] Yang D, Lee JS, Choi CK, Lee HP, Cho SW, Ryu W. Microchannel system for rate-controlled, sequential, and pH-responsive drug delivery. Acta Biomater. 2018; 68:249–260. 10.1016/j.actbio.2017.12.013 29269333

[181] Severino P, da Silva CF, Andrade LN, de Lima Oliveira D, Campos J, Souto EB. Alginate Nanoparticles for Drug Delivery and Targeting. Curr Pharm Des. 2019; 25:1312–1334. 10.2174/1381612825666190425163424 31465282

[182] Tang Xiang YS (2023). Research progress of nanostructured lipid carriers in lung targeted delivery system. China Pharm.

[183] Jain K. Nanobiotechnology-based drug delivery to the central nervous system. Neurodegenerative Diseases. 2007; 4:287–291. 10.1159/000101884 17627131

[184] Ipar VS, Dsouza A, Devarajan PV. Enhancing Curcumin Oral Bioavailability Through Nanoformulations. Eur J Drug Metab Pharmacokinet .2019; 44:459–480. 10.1007/s13318-019-00545-z 30771095

[185] Blasi P, Giovagnoli S, Schoubben A, Ricci M, Rossi C. Solid lipid nanoparticles for targeted brain drug delivery. Adv Drug Deliv Rev. 2007; 59:454–477. 10.1016/j.addr.2007.04.011 17570559

[186] Kumari A, Yadav SK, Yadav SC. Biodegradable polymeric nanoparticles based drug delivery systems. Colloids Surf B Biointerfaces. 2010; 75:1–18. 10.1016/j.colsurfb.2009.09.001 19782542

[187] Gu Wei WC, Liu X (2022). Synthesis of water-soluble paliperidone prodrug and its transdermal study in vitro. Drug Eval Res.

[188] Anselmo AC, Mitragotri S. Nanoparticles in the clinic: An update. Bioeng Transl Med. 2019; 4:10143–10154. 10.1002/btm2.10143 PMC6764803 31572799

[189] Zong-ru G (2022). Clopidogrel, a biological prodrug fortunately discovered. Acta Pharm.

[190] Jiang Yuanqi DY, Chen J (2021). Research progress of targeted preparations of traditional Chinese medicine. Chin Herbal Med.

[191] Su Hui LC (2020). Research progress of targeted preparation in the treatment of rheumatoid arthritis. Med J.

[192] Fang Lin ZP, Zhang Q (2018). Research progress of targeted preparations of adriamycin. Zhongnan Pharm.

[193] Chen Mengzhang LH (2023). Research progress of therapeutic nucleic acid and viral vector vaccine for chronic hepatitis B. Pharm Biotechnol.

[194] Chen Juan PL, Yang X (2022). Analysis of global research and development situation of virus vector vaccine products. China Pharm.

[195] Song Sankong BY, Liang J (2023). Application progress of parallel artificial membrane osmotic model in drug permeability screening of transdermal drug delivery system. China Pharm.

[196] Jin Yuanyuan ZX, Chen J (2022). Research progress of new drug delivery system in transdermal drug delivery. Jilin Trad Chin Med.

[197] Xue Yaqi WZ, Liang P (2022). Progress in molecular pharmaceutics of transdermal preparations of traditional Chinese medicine. J Nanjing Univ Trad Chin Med.

[198] Zhou Ziye GM, Chen P (2023). meta-analysis of the effectiveness and safety of midazolam nasal administration in children's oral diagnosis and treatment. J Clin Stomatol.

[199] Wang Jing CR, Ge Y (2022). Study on the law of nasal administration in ancient Chinese medicine. West Chin Med.

[200] Li Juan LY, Liu Y (2022). Anti-epileptic effect of ginsenoside Rb_1 by nasal administration on pentylenetetrazol-ignited mice. Chin J Exp Trad Med Formulae..

[201] Zeng Yuhui HZ, Lin H (2022). Analysis of the curative effect of pevisone in the treatment of fungal otitis externa. China J Endosc..

[202] Yang Hong ZJ, Ren D (2020). Safety evaluation of volatile oil from Artemisia argyi by external auditory canal in guinea pigs. J Yunnan Coll Trad Chin Med.

[203] Hou S, Gao Y-E, Ma X, Lu Y, Li X, Cheng J, Wu Y, Xue P, Kang Y, Guo M. Tumor microenvironment responsive biomimetic copper peroxide nanoreactors for drug delivery and enhanced chemodynamic therapy. Chemical Engineering Journal. 2021; 416:129–137.

[204] Wang Yue HY, Zhu D (2021). Application of nano-drug delivery system based on polyamide dendrimer prodrug in the treatment of malignant tumor. Zhongnan Pharm.

[205] Zhang Zhigang FX, Lian Lulu. Research progress on regulating the permeability of blood-brain barrier by aromatic resuscitation drugs. 2022; 15:1510–1516.

[206] Li Bonan NG, Sun T (2022). Progress in research and application of exosomes on testicular microenvironment. Progr Biochem Biophys.

[207] Jiang Jianbo SQ, Ding F (2021). Extracellular ion microenvironment regulates sleep awakening. Chin J Clin Pharmacol Ther.

[208] Zhang Kai WK, Xu Y, Xu J, Xu S, Dai K, Qian J, Yang Q (2017). Development of Hawthorn leaf total flavonoids extended-release capsules based on solid dispersion technology and study on the release mechanism of dru. Mod Chin Appl Pharm.

[209] Bang YU, Huang C, Liu M, Zhang H (2023). Systematic evaluation of traditional Chinese medicine nebulized inhalation method combined with conventional western medicine treatment for acute exacerbation of chronic obstructive pulmonary disease. Chin Med Emerg.

[210] Baoolin L. A phase I clinical trial of clinical safety, tolerability, and pharmacokinetics of injectable and haptoglobin liposomes (HK) for the treatment of patients with advanced malignant solid tum. China Drug Clinical Trial Registration and Information Publication Platform. 2017; http://www.chinadrugtrials.org.cn/clinicaltrials.searchlistdetail.dhtml. Accessed 21 Sept 2023.1–4.

[211] Yu Z, Wang D, Qi Y, Liu J, Zhou T, Rao W, Hu K (2023). Autologous-cancer-cryoablation-mediated nanovaccine augments systematic immunotherapy. Mater Horiz.

[212] Lisa Liu GZ, Wang S, Su Z, Yu B, Ma T, Bai Z (2021). Study on the effect of Chinese medicine acupoint application on infrared thermography and clinical efficacy and evaluation of traditional Chinese medicine in middle-aged and elderly patients with thoracic paralytic heartache. Liaoning J Chin Med..

[213] Tingting D. Clinical study on the treatment of chloasma with soluble microneedle patches of "Yu Yan San". Shandong Univ Chin Med. 2022;43:12–15.

[214] WANG Feifan,SONG Yunjia,WU Wenmeng et al. Analysis of the preparation and early drug release of epimedium glycoside/TiO2 nanotube composite coating on pure titanium surface. Chinese Tissue Engineering Research.2016;20(43):6416–6423.

[215] Ying L. Tolerability and pharmacokinetic clinical trial study of single, multiple dose groups, randomized, double-blind, placebo-controlled, sustained-release microspheres of *Staphylococcus**aureus* for Injection. China Drug Clinical Trial Registration and Information Publication Platform. 2014; http://www.chinadrugtrials.org.cn/clinicaltrials.searchlistdetail.dhtml. Accessed 21 Sept 2023, 1–5.

[216] Huaqing W. A phase I study to assess the tolerance and pharmacokinetics of ursolic acid liposomes. Chinese Clinical Trial Registry. 2015; https://www.chictr.org.cn/showproj.html?proj=132682. Accessed 21 Sept 2023,1–6.

[217] Jianchun D. Real world study to evaluate paclitaxel liposome/carboplatin combined with PD-1 monoclonal antibody in the first-line treatment of advanced lung squamous cell carcinoma. Chinese Clinical Trial Registry. 2022; https://www.chictr.org.cn/showproj.html?proj=132682. Accessed 21 Sept 2023,1–4.

[218] Su Y. A phase I clinical trial to evaluate the safety, tolerability, and pharmacokinetic profile of injectable paclitaxel polymeric micelles in Chinese patients with advanced malignant solid tumors. China Drug Clinical Trial Registration and Information Publication Platform. 2021;http://www.chinadrugtrials.org.cn/clinicaltrials.searchlistdetail.dhtml. Accessed 21 Sept 2023,1–5.

